# New Polyether Triterpenoids from *Laurencia viridis* and Their Biological Evaluation

**DOI:** 10.3390/md9112220

**Published:** 2011-11-07

**Authors:** Francisco Cen Pacheco, Janny A. Villa-Pulgarin, Faustino Mollinedo, Manuel Norte Martín, José Javier Fernández, Antonio Hernández Daranas

**Affiliations:** 1 University Institute for Bio-Organic Chemistry “Antonio González” (IUBO), University of La Laguna (ULL), Astrofísico Francisco Sánchez 2, La Laguna, Tenerife 38206, Spain; E-Mail: saiyacen@hotmail.com; 2 Institute of Molecular and Cellular Biology of Cancer, Cancer Research Center, CSIC-University of Salamanca, Campus Miguel de Unamuno, Salamanca E-37007, Spain; E-Mails: jvilla@usal.es (J.A.V.-P.); fmollin@usal.es (F.M.); 3 APOINTECH, Spanish-Portuguese Center for Agriculture Research (CIALE), Scientific Park of the University of Salamanca, C/Rio Duero 12, Villamayor, Salamanca E-37185, Spain

**Keywords:** *Laurencia viridis*, squalene, polyethers, cytotoxic activity

## Abstract

The red seaweed *Laurencia viridis* is a rich source of secondary metabolites derived from squalene. New polyethers, such as iubol (**2**), 22-hydroxy-15(28)- dehydrovenustatriol (**3**), 1,2-dehydropseudodehydrothyrsiferol (**4**), and secodehydrothyrsiferol (**5**) have been isolated and characterized from this alga. The structures were determined through the interpretation of NMR spectroscopic data and the relative configuration was proposed on the basis of NOESY spectrum and biogenetic considerations. All new compounds exhibited significant cytotoxic activity against a panel of cancer cell lines.

## 1. Introduction

Within marine natural products, polyether metabolites encompass a unique class displaying a great diversity of structures and a broad array of bioactivities, acting as enzymatic inhibitors, ion channel blockers, stimulating neurotransmitters release or showing potent cytotoxic activities [1–4]. One important group of squalene-derived polyethers, has been isolated from red algae of the genus *Laurencia* and *Chondria*, (Rhodomelaceae family), sponges of the Axinellidae family, and from some mollusks [3–5]. Undoubtedly, the main source of these metabolites is the red alga *Laurencia viridis*, endemic algae of the Canary Islands that grow on basaltic rocks in the lower intertidal zone at early spring when the coastal temperature is about 18 °C [5,6]. Dehydrothyrsiferol (**1**), the major metabolite of this class, has shown important pharmacologic properties such as potent cytotoxic effects, protein phosphatase type 2A inhibition and integrin antagonist activity [7–11].

**Figure 1:**
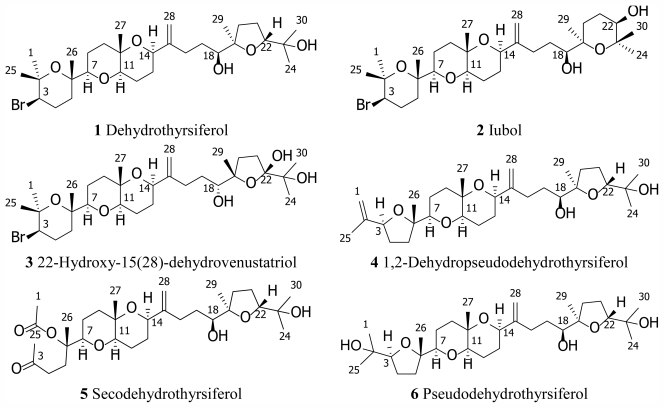
Structures of squalene-derived polyethers isolated from *Laurencia viridis*.

Further investigation of the minor constituents present in the fresh alga *L. viridis* led to the isolation of four novel polyethers, iubol (**2**), 22-hydroxy-15(28)-dehydrovenustatriol (**3**), 1,2-dehydropseudodehydrothyrsiferol (**4**) and secodehydrothyrsiferol (**5**). Herein, we describe the isolation and structure determination of these compounds as well as their cytotoxic effects in several cancer cell lines (Figure 1).

## 2. Results and Discussion

**Figure 2:**
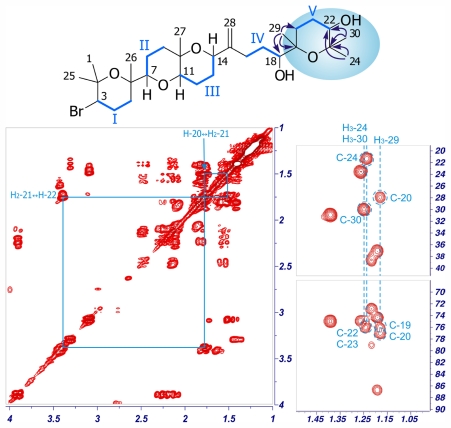
Selected sections of the COSY and HMBC spectra of compound **2**. ^1^H–^1^H spin systems are shown in blue lines, while arrows represent significant HMBC correlations.

Iubol (**2**), was isolated as an amorphous white solid, [α]^25^ _D_ +14.8 (*c* 0.27, CHCl_3_). The molecular formula, C_30_H_51_O_6_Br, determined by ESI-HRMS (*m/z* 609.3679/611.3607, [M + Na]^+^), turned out to be identical to that found for dehydrothyrsiferol (**1**) [7,12]. The structure of **2** was mainly determined from the analysis of its NMR data. Interpretation of its ^1^H and ^13^C NMR spectra together with the examination of the HSQC experiment showed that **2** consists of seven methyls, eleven methylenes, six methines and six quaternary carbons. Analysis of the ^1^H–^1^H COSY spectra enabled us to set five independent proton-proton spin systems, in a similar way to that found in other polyethers isolated from *L. viridis* [5,7,8]. Specifically, the fragment C-1→C-18 of **2** closely resembles that of **1**, with the presence of the same four spin systems: Fragment I [H-3 (δ_H_ 3.89), H_2_-4 (δ_H_ 2.10/2.24), H_2_-5 (δ_H_ 1.53/1.80)]; Fragment II [H-7 (δ_H_ 3.08), H_2_-8 (δ_H_ 1.45/1.72), H_2_-9 (δ_H_ 1.50/1.77)]; Fragment III [H-11 (δ_H_ 3.42), H_2_-12 (δ_H_ 1.63/1.78), H_2_-13 (δ_H_ 1.82/2.03), H-14 (δ_H_ 4.27)] and Fragment IV [H_2_-16 (δ_H_ 2.12/2.46), H_2_-17 (δ_H_ 1.38), H-18 (δ_H_ 3.30)] (Figure 2; Table 1). On the other hand, notable differences were found towards the terminal moiety of the molecule (C-19→C-24), where the COSY spectrum revealed coupling between the characteristic proton H-22 (δ_H_ 3.38) and H_2_-21 (δ_H_ 1.75) and these sequentially to both H_2_-20 (δ_H_ 1.50/1.79). Finally, the structure of **2** was unambiguously assigned by the HMBC correlations. Thus, significant correlations between methyl H_3_-29 (δ_H_ 1.18) with C-18 (δ_C_ 77.0), C-19 (δ_C_ 76.1) and C-20 (δ_C_ 27.9), as well as those of methyls H_3_-24 (δ_H_ 1.24) and H_3_-30 (δ_H_ 1.25) with C-22 (δ_C_ 75.2) and C-23 (δ_C_ 75.7), were observed (Figure 2).

**Figure 3:**
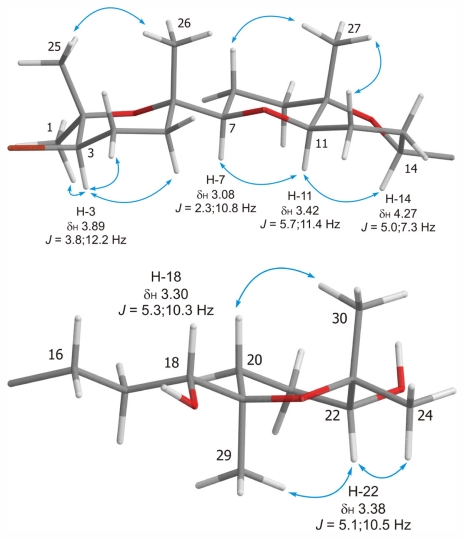
Significant dipolar correlations observed in NOESY experiments in **2**.

The high coincidence between the ^1^H and ^13^C NMR data of the C-1→C-14 fragment in **2** with those observed for **1** indicated that both compounds share the same relative configuration at C-3, C-6, C-7, C-10, C-11 and C-14 (Table 1). This conclusion was further supported by the analysis of the NOESY experiment, where identical correlations between **1** and **2** were observed. In particular, those connecting H-3 with H_3_-1 (δ_H_ 1.26), H-4α (δ_H_ 2.10) and H-5α (δ_H_ 1.53); H_3_-25 (δ_H_ 1.39) with H_3_-26 (δ_H_ 1.20) as well as H-11 with H-7 and H-14 together with those of H_3_-27 (δ_H_ 1.22) with H-8β (δ_H_ 1.45) and H-12β (δ_H_ 1.63) secured the relative configuration of this fragment. With regard to the C-18→C-24 fragment, when H-22 (δ_H_ 3.38, dd, *J* = 5.1, 10.5 Hz) was selectively irradiated in the 1D-*g*NOESYexperiment, only H_3_-29 and H_3_-24 showed enhancements indicating that all protons are on the same side of the tetrahydropyran ring. From these coupling constant values and NOESY correlations we concluded that only a twist-boat conformation with 19R*, 22R* relative configuration is possible for the D ring, as shown in Figure 3.

**Figure 4:**
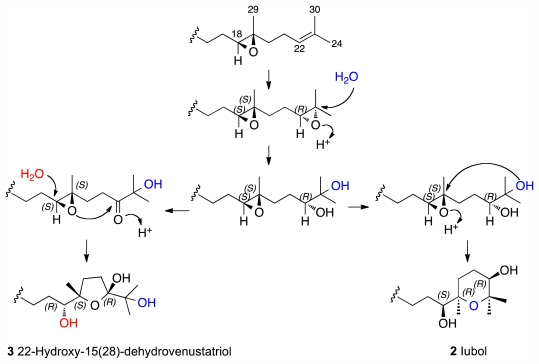
Biogenetic proposal for the C-16→C-24 moieties of compounds **2** and **3**.

From a biogenetic viewpoint, most of the polyoxygenated squalene-derived ethers isolated from *Laurencia* species seem to arise from a common precursor: the (10*R*,11*R*)-squalene 10,11-epoxide isolated from *L. okamurai*. This compound can evolve to (6*S*,7*S*,10*R*,11*R*,14*R*,15*R*,18*S*,19*S*)-squalene tetraepoxide as a common intermediate [5]. Therefore, the new tetrahydropyran ring found in **2** could be biosynthesised from the diepoxide fragment 18*S*, 19*S*, 22*R* by protonation of the (20*R*)-epoxide followed by hydroxylation at C-23 yielding 18*S*, 19*S*-epoxy-20*R*-hydroxy as intermediate. The subsequent cyclization process with configuration inversion at C-19 would give the 18S*, 19R*, 22R* relative configuration as illustrated in Figure 4. Importantly, this hypothesis is consistent with the experimental data used to proffer the relative configuration of C-19 and C-22 offering simultaneously a solution for the configuration of C-18.

**Figure 5:**
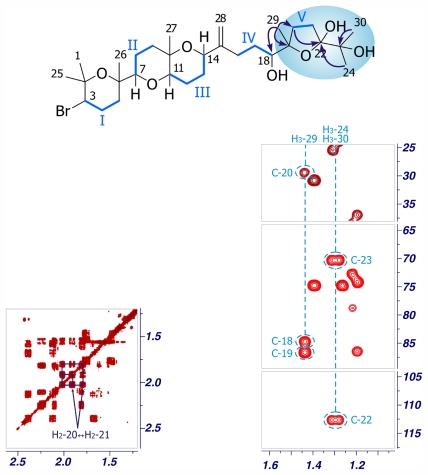
Selected sections of the COSY and HMBC spectra of compound **3**. ^1^H–^1^H spin systems are represented with blue lines and arrows represent significant HMBC correlations.

22-Hydroxy-15(28)-dehydrovenustatriol (**3**), was obtained as an amorphous white solid, [α]^25^ _D_ +15.2 (*c* 0.07, CHCl_3_). The molecular formula of **3**, C_30_H_51_O_7_Br, deduced by ESI-HRMS (*m/z* 625.2695/627.2723, [M + Na]^+^), indicated the presence of an additional oxygen atom with respect to dehydrothyrsiferol (**1**) [7,12]. Comparison of the ^1^H and ^13^C NMR data of **3** with those reported for **1** clearly revealed that **3** shares the same carbon skeleton with **1**, but contains an additional oxygen atom at the C-19→C-24 moiety (Table 2). Thus, a detailed analysis of the ^1^H–^1^H COSY and HSQC spectra confirmed the existence of the same four ^1^H–^1^H spin systems (Fragments I–IV) observed in the C-1→C-18 portion of **1** (Figure 5). However, in contrast with **1**, the terminal moiety of the molecule contains only a four-proton spin system formed by H_2_-20 (δ_H_ 2.00) and H_2_-21 (δ_H_ 1.80/1.91), missing the characteristic H-22 signal. Finally, we used the HMBC experiment to connect the new and characteristic ^13^C signal of C-22 (δ_C_ 112.5) with H_2_-20 (δ_H_ 2.00), H_3_-24 (δ_H_ 1.29) and H_3_-30 (δ_H_ 1.31). In addition those correlations observed between H_3_-29 (δ_H_ 1.44) and C-18 (δ_C_ 84.8), C-19 (δ_C_ 86.9) and C-20 (δ_C_ 29.3) allowed us to build up the structure of the C-18→C-24 portion of **3**.

Comparison of the ^3^*J*_H–H_ coupling constants, ^1^H and ^13^C chemical shifts together with a detailed analysis of the NOESY experiment established the relative configuration of the C-1→C-14 fragment in **3** as identical to dehydrothyrsiferol (**1**). However, the absence of NOE correlations between H_3_-29 with H_3_-24 and H_3_-30, excluded any definitive conclusion although it clearly suggests a *trans* configuration for this oxolane ring. The relative configuration of this moiety can be proposed making use of a biogenetic hypothesis. Thus, the presence of a hemiacetal group at C-22 can be rationalized via an intermediate with a carbonyl group at C-22 and by the subsequent cyclization as represented in Figure 4. Again, assuming that the biogenesis starts with a common precursor for all the oxaqualenoids isolated from *Laurencia*, the most plausible configuration for the stereogenic centers of this fragment should be 18R*, 19S* and 22R*.

**Figure 6:**
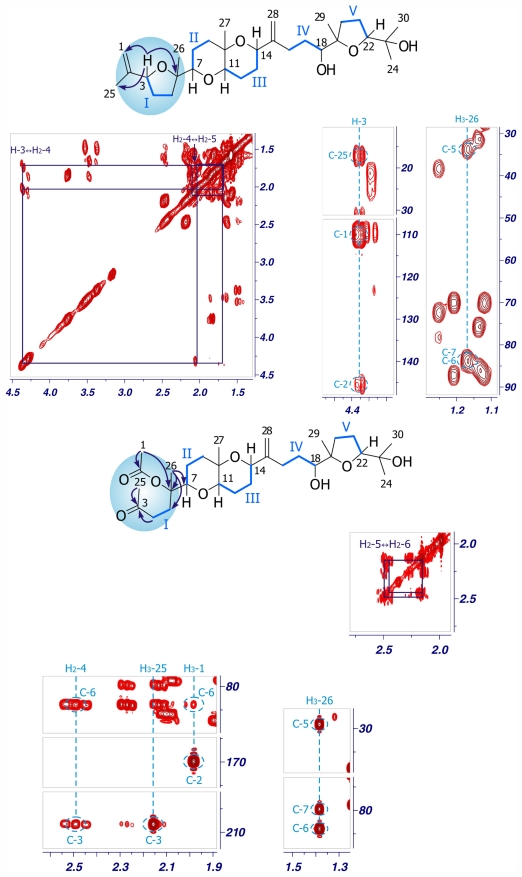
Selected sections of the COSY and HMBC spectra of compounds **4** and **5**. Blue lines represent ^1^H–^1^H spin systems while arrows indicate relevant HMBC correlations.

**Figure 7:**
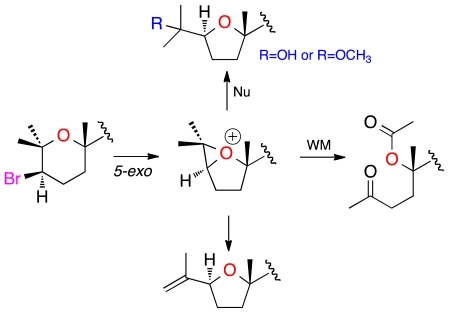
Plausible biogenesis pathway for metabolites showing rings A modifications.

Two additional related compounds, 1,2-dehydropseudodehydrothyrsiferol (**4**) and secodehydrothyrsiferol (**5**) were isolated in this study. Analysis of their ESI-HRMS spectra allowed us to establish their molecular formulas as C_30_H_50_O_6_ and C_30_H_50_O_8_ respectively. Comparison of the ^1^H and ^13^C chemical shifts of 1,2-dehydropseudodehydrothyrsiferol (**4**) with other related compounds revealed great similarities with those reported for pseudodehydrothyrsiferol (**6**) except at the C-1→C-3 portion of the molecule (Tables 2 and 3). In fact, analysis of the 2D NMR spectra led us to the same carbon skeleton. However, the observation of HMBC correlations between the methylene protons H_2_-1 (δ_H_ 4.77/4.99) with C-2 (δ_C_ 145.3), C-3 (δ_C_ 83.2) and C-25 (δ_C_ 17.2) clearly indicated that the differences between **4** and **6** were due to the dehydration of the hydroxyl group attached to C-2 in **6**, that yielded an exocyclic double bond at C-1→C-2 in **4** (Figure 6). With regard to secodehydrothyrsiferol (**5**), the NMR data showed changes with respect to **6** around the C-1→C-6 fragment. The ^1^H NMR spectrum revealed the existence of two new methyl groups as well as one methylene located at α-carbonyl positions. In addition, two new carbonyl groups (one ketone and one ester) were easily detected from the ^13^C NMR spectrum. Therefore, the structure of **5** was determined on the basis of the connectivity observed between H_2_-4 (δ_H_ 2.50) and H_2_-5 (δ_H_ 2.16/2.26) in the COSY experiment together with the HMBC correlations of H_3_-25 (δ_H_ 2.15) and H_2_-4 with C-3 (δ_C_ 208.5), those of H_3_-1 (δ_H_ 1.98) with C-2 (δ_C_ 170.1) and C-6 (δ_C_ 84.1) as well as those of H_3_-26 (δ_H_ 1.39) with C-5 (δ_C_ 29.3), C-6 (δ_C_ 84.1) and C-7 (δ_C_ 80.0). Comparison of the ^1^H and ^13^C chemical shifts, ^3^*J*_H–H_ coupling constants, together with an analysis of the ROESY experiments of **4** and **5** showed very similar results compared to those of compound **6**, and therefore the same relative configuration is proposed for the new compounds. An important feature in **4** and **5** is the absence of the bromine atom in ring A as it is common in the thyrsiferol or venustatriol series. Thus, the biogenetic origin of both compounds could be rationalized by attack of the oxane oxygen to the C-3 position that bears a bromine atom via a *5-exo* process, giving an oxonium ion intermediate that would evolve as shown in Figure 7 to yield both metabolites [5]. The biogenetic origin of **5** could be rationalized by a Wagner-Meerwein rearrangement. These rearrangements have been confirmed experimentally in many cases and are almost always consistent with the participation of an oxonium ion intermediate [13,14]. In order to confirm the previous proposal, we induced this rearrangement in dehydrothyrsiferol (**1**). Therefore, **1** was treated with AgNO_3_ using either methanol:water (1:1) or anhydrous acetonitrile as solvents. In the first case, using a methanol:water (1:1) mixture, the reaction yielded pseudodehydrothyrsiferol (**6**) as well as the methoxy derivatives at C-2 and C-3 positions. The relative yields of this reaction could be modulated changing the methanol:water ratio. On the other hand, when an aprotic solvent was used, three compounds showing *m/z* of 506 were identified by LC-MS although the main product was 1,2-dehydropseudodehydrothyrsiferol (**4**) [15,16].

**Figure 8:**
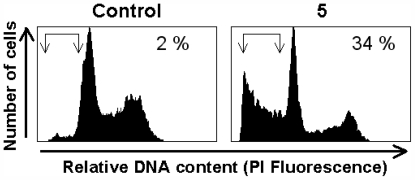
Induction of apoptosis by secodehydrothyrsiferol (**5**) in T-cell leukemia cells. Jurkat cells were treated with 20 μM of **5** for 48 h. Afterwards apoptosis was quantitated as the percentage of cells in the sub-G_1_/G_0_ region (arrows) in cell cycle analysis by flow cytometry. Untreated control cells were run in parallel. The percentage of apoptotic cells is indicated in each histogram.

This class of polyether squalene natural products shows a variety of biological properties where its cytotoxicity against breast cancer cells is highly significant [5,9,17]. Therefore, the *in vitro* cytostatic activity of **1**–**5** was assessed by XTT assays [18], using several human cancer cell lines, including Jurkat (human T-cell acute leukemia), MM144 (human multiple myeloma), HeLa (human cervical carcinoma), and CADO-ES1 (human Ewing’s sarcoma). As shown in Table 4, Jurkat leukemic cells were the most sensitive cells to the tested polyether compounds. In particular, iubol (**2**), 22-hydroxy-15(28)-dehydrovenustatriol (**3**), and secodehydrothyrsiferol (**5**) showed the highest effectiveness against this cell line (IC_50_, 2.0–3.5 μM). The capacity of the above compounds to inhibit cell proliferation was likely due to their ability to induce apoptosis, as assessed by the appearance of a sub-G_1_/G_0_ subpopulation in cell cycle analysis, indicative of DNA breakdown [19], following incubation of Jurkat cells with **5** (Figure 8).

Interestingly, the substitution of an H atom in **1** by a hydroxyl group in **3** at C-22 highly potentiated the cytostatic activity (Table 4). It is also noteworthy that all the above compounds were active against the CADO-ES1 human Ewing’s sarcoma cell line (Table 4) in the range of 10–12 μM, suggesting that this type of compound could be suitable in the search for therapeutic lead compounds against both sarcoma and T-cell leukemia malignancies. Doxorubicin, used as a positive control rendered IC_50_ values in the order of 10^−7^–10^−8^ M (data not shown).

## 3. Experimental Section

### 3.1. General Experimental Procedures

Optical rotation was determined on a Perkin-Elmer 241 polarimeter. IR spectra were measured on a Bruker IFS55 spectrometer. The NMR spectra were obtained with a Bruker 600 Advance instruments. Chemical shifts (δ) are reported in ppm and referenced to the residual peak of CHCl_3_ at 7.26 ppm and 77.0 ppm for ^1^H and ^13^C, respectively. The couplings constants are given in Hz. ESI-HRMS was performed on a VG AutoSpec FISON spectrometer. HPLC was carried out with a LKB 2248 system equipped with a differential diffractometer detector. Silica gel CC and TLC were performed on Silica gel Merck 60 G. TLC plates were visualized by spraying with H_2_SO_4_/H_2_O/AcOH (1:4:20) and heating.

### 3.2. Plant Material

The specimens of *Laurencia viridis* were collected in March 2008 in Callao Salvaje, Paraiso Floral, Adeje (Tenerife, Canary Island). A voucher specimen was deposited at the herbarium of the La Laguna University, Department of Vegetal Biology, Botany, Tenerife.

### 3.3. Extraction and Chromatographic Separation

The fresh alga was extracted with a 1:1 mixture of CHCl_3_:MeOH at room temperature. The extract was evaporated *in vacuo* to leave dark-green viscous oil (83.0 g, 1.5% dry weight). The crude extract was chromatographed on a Sephadex LH-20 column using CHCl_3_:MeOH (1:1). Fractions that were similar in composition as shown by TLC were combined to give four fractions. The second fraction (53.4 g) was further separated by silica gel column eluted with increasing concentrations of EtOAc in *n*-hexane. The third fraction (4.43 g) was chromatographed by a medium pressure silica gel chromatography Lobar LiChroprep-Si60with CH_2_Cl_2_:acetone (8:2) as eluent. Final purification was carried out by HPLC employing μ-Porasil column and using *n*-Hex:EtOAc, *n*-Hex:acetone and CH_2_Cl_2_:acetone in different proportions affording the pure new compounds, iubol (**2**) (2.70 mg), 22-hydroxy-15(28)-dehydrovenustatriol (**3**) (0.60 mg), 1,2-dehydropseudodehydrothyrsiferol (**4**) (5.13 mg) and secodehydrothyrsiferol (**5**) (0.80 mg).

#### Iubol (**2**)

Amorphous white solid; [α]^25^ _D_ +14.8 (*c* 0.27, CHCl_3_); IRν_max_ (CHCl_3_) 3422, 2950, 2868, 1649, 1457, 1377 and 1098 cm^−1^; ESI-MS *m/z* 611, 609, 507, 506, 445, 443, 207 and 205; ESI-HRMS *m/z* 611.3607 (Calcd. for C_30_H_51_O_6 81_BrNa, 611.3610, [M + Na]^+^); ^1^H and ^13^C data NMR see Table 1.

#### 22-Hydroxy-15(28)-dehydrovenustatriol (**3**)

Amorphous white solid; [α]^25^ _D_ +15.2 (*c* 0.07, CHCl_3_); IRν_max_(CHCl_3_) 3494, 2956, 2872, 1465, 1381 and 1102 cm^−1^; ESI-MS *m/z* 627, 625, 609, 607, 413, 301 and 277; ESI-HRMS *m/z* 627.2723 (Calcd. for C_30_H_51_O_7 81_BrNa, 627.2695, [M + Na] ^+^); ^1^H and ^13^C data NMR see Table 2.

#### 1,2-Dehydropseudodehydrothyrsiferol (**4**)

Amorphous white solid; [α]^25^ _D_ −4.9 (*c* 0.51, CHCl_3_); IRν_max_ (CHCl_3_) 3396, 2971, 2870, 1453, 1375 and 1093 cm^−1^; ESI-MS *m/z* 506, 488, 380, 363, 209, 143 and 125; ESI-HRMS *m/z* 506.3627 (Calcd. for C_30_H_50_O_6_, 506.3607, [M]^+^); ^1^H and ^13^C data NMR see Table 2.

#### Secodehydrothyrsiferol (**5**)

Amorphous white solid; [α]^25^ _D_ +2.5 (*c* 0.08, CHCl_3_); IRν_max_ (CHCl_3_) 2967, 2869, 1724, 1375 and 1096 cm^−1^; ESI-MS *m/z* 562, 561, 547, 413, 301 and 236; ESI-HRMS *m/z* 561.3407 (Calcd. for C_30_H_50_O_8_Na, 561.3403, [M + Na]^+^); ^1^H and ^13^C data NMR see Table 3.

### 3.4. Chemical Correlations

To a solution of dehydrothyrsiferol (**1**) (5.0 mg, 8.5 μmol) in anhydrous acetonitrile (1 mL) was added AgNO_3_ (2.2 mg, 12.7 μmol). The solution was stirred for one hour at 40 °C and the resulting material was filtered off and concentrated under reduced pressure. The crude material was chromatographed in HPLC (μ-Porasil column, *n*-Hex/AcOEt/MeOH14:5:1, flow rate 1 mL/min) to afford the 1,2-dehydropseudodehydrothyrsiferol 1.0 mg, (2.0 μmol) [14]. On other hand*,* To a mixture of dehydrothyrsiferol (**1**) (10 mg, 17 μmol) in 1 mL MeOH:H_2_O (1:1) was added AgNO_3_ (4.4 mg, 25.4 μmol) and the reaction solution was stirred for 30 min a room temperature. The resulting solution was filtered off and the 2 mL of MeOH:H_2_O was removed *in vacuo*. The crude oil was purified in HPLC (μ-Porasil column, CH_2_Cl_2_/MeOH 19:1, flow rate 0.5 mL/min) afforded 4.8 mg (9.1 μmol) of pseudodehydrothyrsiferol (**6**) 3.5 mg (6.5 μmol) of 2-methoxypseudodehydrothyrsiferol and 0.7 mg (1.3 μmol) of 2-dehydroxy-3-methoxypseudodehydrothyrsiferol [15].

### 3.5. Cell Culture

Jurkat (human T-cell acute leukaemia), MM144 (human multiple myeloma), HeLa (human cervical carcinoma), and CADO-ES1 (human Ewing’s sarcoma) cells were cultured in RPMI-1640 (Jurkat, MM144, CADO-ES1) or DMEM (HeLa) culture medium containing 10% (v/v) heat-inactivated fetal bovine serum (FBS), 2 mM l-glutamine, 100 U/mL penicillin, and 100 μg/mL streptomycin at 37 °C in air containing 95% humidity and 5% CO_2_. Cells were periodically tested for Mycoplasma infection using the MycoAlert^©^ Mycoplasma detection kit (Lonza, Basel, Switzerland) as well as the Venor^©^GeM Advance Mycoplasma PCR detection Kit (Minerva Biolabs, Berlin, Germany), and found to be negative.

### 3.6. Cell Growth Inhibition Assay

The effect of compounds **1**–**5** in the proliferation of human tumor cell lines (cytostatic activity) was determined as previously described by using the XTT (sodium 3′-[1-(phenylaminocarbonyl)-3,4- tetrazolium]-bis(4-methoxy-6-nitro)benzenesulfonic acid hydrate) cell proliferation kit (Roche Molecular Biochemicals, Mannheim, Germany) as previously described [18]. Cells (1.5–5.0 × 10^3^ in 100 μL) were incubated in RPMI-1640 (Jurkat, MM144, CADO-ES-1) or DMEM (HeLa) culture medium containing 10% heat-inactivated FBS, in the absence and in the presence of the indicated compounds at a concentration range of 10^−4^ to 10^−9^ M, in 96-well flat-bottomed microtiter plates, and following 72 h of incubation at 37 °C in a humidified atmosphere of air/CO_2_ (19/1) the XTT assay was performed. Measurements were done in triplicate, and each experiment was repeated three times. The IC_50_ (50% inhibitory concentration) value, defined as the drug concentration required to cause 50% inhibition in the cellular proliferation with respect to the untreated controls, was determined for each compound. Data are shown as means ± standard deviation (SD) of three independent experiments, each performed in triplicate.

### 3.7. Apoptosis Assay

Quantitation of apoptotic cells was calculated by fluorescence flow cytometry as the percentage of cells in the sub-G_1_/G_0_ region (hypodiploidy) in cell cycle analysis as previously described [19], by using a Becton Dickinson FACSCalibur flow cytometer (San Jose, CA, USA).

## Supplementary Material



## Figures and Tables

**Table 1:** NMR data (600 MHz, CDCl_3_, 298 K) for dehydrothyrsiferol (**1**) and iubol (**2**).

	Dehydrothyrsiferol (1)				Iubol (2)			

Position	δ_C_, mult.		δ_H_ (*J* in Hz)	HMBC a	δ_C_, mult.		δ_H_ (*J* in Hz)	HMBC a
**1**	31.0,	CH_3_	1.27, s	2, 3, 25	31.0,	CH_3_	1.26, s	2, 3, 25
**2**	74.9,	C			74.9,	C		
**3**	59.0,	CH	3.89, dd (4.1, 12.6)	1, 2, 4, 5, 25	59.1,	CH	3.89, dd (3.8, 12.2)	1, 2, 5, 25
**4**	28.2,	CH_2_	2.13(α), m 2.26(β), m	2, 3, 5, 6	28.3,	CH_2_	2.10(α), m 2.24(β), m	2, 3, 5, 6
**5**	37.1,	CH_2_	1.52(α), m 1.80(β), m	3, 4, 6, 7	37.1,	CH_2_	1.53(α), m 1.80(β), m	3, 4, 6, 7
**6**	74.4,	C			74.3,	C		
**7**	86.7,	CH	3.08, dd (2.5, 11.0)	6, 8, 9, 11	86.6,	CH	3.08, dd (2.3, 10.8)	6, 8, 9, 11
**8**	22.9,	CH_2_	1.49(β), m 1.76(α), m	7, 10	23.0,	CH_2_	1.45(β), m 1.72(α), m	7, 10
**9**	38.7,	CH_2_	1.44(α), m 1.77(β), m	7, 10, 11	38.7,	CH_2_	1.50(α), m 1.77(β), m	7, 10, 11
**10**	72.9,	C			72.9,	C		
**11**	78.9,	CH	3.43, dd (5.7, 11.3)	7, 9, 10, 12, 13	79.1,	CH	3.42, dd (5.7, 11.4)	7, 10, 13
**12**	21.8,	CH_2_	1.60(β), m 1.81(α), m	10, 11, 14	21.8,	CH_2_	1.63(β), m 1.78(α), m	10, 11, 14
**13**	26.6,	CH_2_	1.86(β), m 2.11(α), m	11, 14	26.3,	CH_2_	1.82(β), m 2.03(α), m	11, 14
**14**	72.5,	CH	4.28, dd (4.0, 8.1)	10, 15, 16, 28	72.4,	CH	4.27, dd (4.8, 7.3)	15, 16, 28
**15**	151.3,	C			151.3,	C		
**16**	29.9,	CH_2_	2.12, m 2.43, m	14, 15, 28, 18	29.9,	CH_2_	2.12, m 2.46, m	14, 15, 28
**17**	30.3,	CH_2_	1.45, m 1.63, m	18, 19	28.2,	CH_2_	1.38	18, 19
**18**	76.2,	CH	3.52, dd (1.7, 10.4)	16, 17, 19, 20	77.0,	CH	3.30, dd (1.3, 10.5)	16, 19, 20
**19**	86.0,	C			76.1,	C		
**20**	31.7,	CH_2_	1.60, m 2.12, m	18, 19, 22	27.9,	CH_2_	1.50, m 1.79, m	18, 19
**21**	26.3,	CH_2_	1.84, m	19, 22	24.7,	CH_2_	1.75, m	22
**22**	87.6,	CH	3.76, dd (6.0, 9.9)	19, 23, 24, 30	75.2,	CH	3.38, dd (5.1, 10.5)	23, 24, 30
**23**	70.5,	C			75.7,	C		
**24**	24.0,	CH_3_	1.12, s	22, 23, 30	21.4,	CH_3_	1.24, s	22, 23, 30
**25**	23.6,	CH_3_	1.39, s	1, 2, 3	23.6,	CH_3_	1.39, s	1, 2, 3
**26**	20.1,	CH_3_	1.20, s	5, 6, 7	20.0,	CH_3_	1.20, s	5, 6, 7
**27**	19.4,	CH_3_	1.23, s	9, 10, 11	19.3,	CH_3_	1.22, s	9, 10, 11
**28**	109.8,	CH_2_	4.88, bs 5.05, bs	14, 15, 16	109.7,	CH_2_	4.86, bs 5.03, bs	14, 15, 16
**29**	23.7,	CH_3_	1.14, s	18, 19, 20	22.4,	CH_3_	1.18, s	18, 19, 20
**30**	27.7,	CH_3_	1.21, s	22, 23, 24	30.0,	CH_3_	1.25, s	22, 23, 24

a HMBC correlations, optimized for 6 Hz, are from proton(s) stated to the indicated carbon.

**Table 2:** NMR spectroscopic data (600 MHz, CDCl_3_, 298 K) for 22-hydroxy-15(28)- dehydrovenustatriol (**3**) and 1,2-dehydropseudodehydrothyrsiferol (**4**).

	22-Hydroxy-15(28)dehydrovenustatriol (3)				1,2-Dehydropseudodehydrothyrsiferol (4)			

Position	δ_C_, mult.		δ_H_ (*J* in Hz)	HMBC a	δ_C_, mult.		δ_H_ (*J* in Hz)	HMBC a
**1**	31.0,	CH_3_	1.27, s	2, 3, 25	110.0,	CH_2_	4.77, bs 4.99, bs	2, 3, 25
**2**	74.7,	C			145.3,	C		
**3**	59.0,	CH	3.89, dd (3.9, 12.5)	1, 2, 5, 25	83.2,	CH	4.36, dd (6.1,8.7)	1, 2, 4, 25
**4**	28.2,	CH_2_	2.11(α), m 2.25(β), m	2, 3, 5, 6	30.9,	CH_2_	1.70, m 2.04, m	3, 6
**5**	36.9,	CH_2_	1.52(α), m 1.81(β), m	3, 4, 6, 7, 26	34.2,	CH_2_	1.62, m 2.10, m	6, 7, 26
**6**	74.3,	C			84.1,	C		
**7**	86.4,	CH	3.08, dd (2.2, 11.2)	6, 9, 11, 26	83.7,	CH	3.37, dd (2.7,11.0)	6, 9, 11, 26
**8**	22.7,	CH_2_	1.47(β), m 1.75(α), m	7, 10	24.6,	CH_2_	1.47(β), m 1.65(α), m	6, 7, 10
**9**	38.5,	CH_2_	1.53(α), m 1.78(β), m	7, 10, 11	38.4,	CH_2_	1.56(β), m 1.80(α), m	10, 11
**10**	73.0,	C			72.4,	C		
**11**	78.8,	CH	3.42, dd (5.7, 11.3)	7, 10, 13, 27	78.6,	CH	3.48, dd (5.5,11.6)	9, 10, 13, 27
**12**	21.7,	CH_2_	1.61(β), m 1.81(α), m	10, 11, 14	21.4,	CH_2_	1.66(β), m 1.82(α), m	10, 11
**13**	26.6,	CH_2_	1.85(β), m 2.10(α), m	11, 14	26.4,	CH_2_	1.85(β), m 2.09(α), m	11, 14, 15
**14**	72.5,	CH	4.26, dd (4.4, 7.4)	15, 16, 28	72.3,	CH	4.28, dd (4.0,7.7)	12, 15, 16, 28
**15**	151.0,	C			151.0,	C		
**16**	29.9,	CH_2_	2.12, m 2.39, m	14, 15, 28	29.3,	CH_2_	2.18, m 2.46, m	15, 28
**17**	29.5,	CH_2_	1.57, m 1.68, m	18, 19	29.7,	CH_2_	1.48, m 1.64, m	15, 18
**18**	84.8,	CH	3.55, m	16, 19, 20, 29	76.0,	CH	3.52, d (9.2)	17, 19, 20, 29
**19**	86.9,	C			86.1,	C		
**20**	29.3,	CH_2_	2.00, m	18, 19	31.3,	CH_2_	1.57, m 2.08, m	18, 19
**21**	32.0,	CH_2_	1.80, m 1.91, m	19, 22	26.3,	CH_2_	1.84, m	19, 22
**22**	112.5,	C			87.4,	CH	3.76, dd (5.8,10.2)	21, 23, 24, 30
**23**	70.4,	C			70.3,	C		
**24**	25.2,	CH_3_	1.29, s	22, 23, 24	23.7,	CH_3_	1.12, s	22, 23, 30
**25**	23.3,	CH_3_	1.40, s	1, 2, 3	17.2,	CH_3_	1.70, s	1, 2, 3
**26**	19.7,	CH_3_	1.20, s	5, 6, 7	22.7,	CH_3_	1.17, s	5, 6, 7
**27**	19.1,	CH_3_	1.22, s	9, 10, 11	19.5,	CH_3_	1.26, s	9, 10, 11
**28**	109.4,	CH_2_	4.86, bs 5.05, bs	14, 15, 16	109.6,	CH_2_	4.88, bs 5.05, bs	14, 15, 16
**29**	17.5,	CH_3_	1.44, s	18, 19, 20	23.6,	CH_3_	1.14, s	18, 19, 20
**30**	23.6,	CH_3_	1.31, s	22, 23, 30	27.5,	CH_3_	1.21, s	22, 23, 24

a HMBC correlations, optimized for 6 Hz, are from proton(s) stated to the indicated carbon.

**Table 3:** NMR spectroscopic data (600 MHz, CDCl_3_, 298 K) for secodehydrothyrsiferol (**5**) and 1,2-pseudodehydrothyrsiferol (**6**).

	Secodehydrothyrsiferol (5)				Pseudodehydrothyrsiferol (6)			

Position	δ_C_, mult.		δ_H_ (*J* in Hz)	HMBC a	δ_C_, mult.		δ_H_ (*J* in Hz)	HMBC a
**1**	22.2,	CH_3_	1.98, s	6, 25	24.0,	CH_3_	1.11, s	2, 3, 25
**2**	170.1,	C			70.6,	C		
**3**	208.5,	C			86.7,	CH	3.76, dd (5.8, 9.1)	1, 2, 4, 5, 25
**4**	38.1,	CH_2_	2.50, m	3, 5, 6	26.3,	CH_2_	1.84, m	3, 6
**5**	29.3,	CH_2_	2.16, m 2.26, m	3, 4, 6, 7, 26	35.2,	CH_2_	1.66, m 2.04, m	6, 7, 26
**6**	84.1,	C			84.0,	C		
**7**	80.0,	CH	3.93, dd (3.9, 9.9)	5, 6, 9, 11, 26	84.0,	CH	3.32, dd (2.6, 11.4)	6, 9, 11, 26
**8**	23.6,	CH_2_	1.56, m	7, 10	24.5,	CH_2_	1.51(β), m 1.66(α), m	7, 10
**9**	38.6,	CH_2_	1.57(β), m 1.81(α), m	10, 11, 27	38.7,	CH_2_	1.57(β), m 1.81(α), m	10, 11, 27
**10**	72.5,	C			72.8,	C		
**11**	79.0,	CH	3.46, dd (5.6, 11.4)	9, 10, 13, 27	78.9,	CH	3.46, dd (5.6, 11.7)	9, 10, 13, 27
**12**	21.7,	CH_2_	1.63(β), m 1.83(α), m	10, 11	21.8,	CH_2_	1.65(β), m 1.84(α), m	10, 11
**13**	26.2,	CH_2_	1.84(β), m 2.05(α), m	11, 14, 15	26.4,	CH_2_	1.85(β), m 2.08(α), m	11, 14, 15
**14**	72.5,	CH	4.28, dd (3.8, 7.5)	13, 15, 16, 28	72.5,	CH	4.29, dd (4.2, 7.1)	13, 15, 16, 28
**15**	151.1,	C			151.3,	C		
**16**	29.5,	CH_2_	2.16, m 2.45, m	15, 28	29.7,	CH_2_	2.20, m 2.46, m	15, 28
**17**	29.9,	CH_2_	1.46, m 1.64, m	15, 18	29.9,	CH_2_	1.48, m 1.64, m	15, 18
**18**	76.2,	CH	3.52, dd (1.4, 10.4)	19, 20, 29	76.2,	CH	3.53, dd (1.5, 10.8)	17, 19, 20, 29
**19**	86.1,	C			86.1,	C		
**20**	31.5,	CH_2_	1.57, m 2.10, m	18, 19	31.6,	CH_2_	1.58, m 2.10, m	18, 19
**21**	26.6,	CH_2_	1.84, m	19, 22	26.5,	CH_2_	1.83, m	19, 22
**22**	87.6,	CH	3.76, dd (5.8, 10.1)	23, 24, 30	87.6,	CH	3.76, dd (6.5, 9.8)	21, 23, 24, 30
**23**	70.4,	C			70.4,	C		
**24**	23.9,	CH_3_	1.13, s	22, 23, 30	23.9,	CH_3_	1.13, s	22, 23, 30
**25**	29.9,	CH_3_	2.15, s	3	27.5,	CH_3_	1.19, s	1, 2, 3
**26**	20.0,	CH_3_	1.39, s	5, 6, 7	22.7,	CH_3_	1.14, s	5, 6, 7
**27**	19.5,	CH_3_	1.25, s	9, 10, 11	19.4,	CH_3_	1.25, s	9, 10, 11
**28**	110.0,	CH_2_	4.89, bs 5.05, bs	14, 15, 16	109.9,	CH_2_	4.89, bs 5.05, bs	14, 15, 16
**29**	23.8,	CH_3_	1.15, s	18, 19, 20	23.7,	CH_3_	1.14, s	18, 19, 20
**30**	27.7,	CH_3_	1.22, s	22, 23, 24	27.7,	CH_3_	1.21, s	22, 23, 24

a HMBC correlations, optimized for 6 Hz, are from proton(s) stated to the indicated carbon.

**Table 4:** *In vitro* growth inhibitory activity of polyether compounds **1**–**5** on tumor cells.

Compound	IC_50_ (μM)			

	Jurkat	MM144	HeLa	CADO-ES1
**1**	13.5 ± 1.8	21.5 ± 2.1	34.5 ± 3.2	12.0 ± 1.4
**2**	3.5 ± 0.4	13.0 ± 1.9	27.0 ± 2.6	11.0 ± 1.5
**3**	2.0 ± 0.2	ND	2.9 ± 0.5	ND
**4**	15.5 ± 2.8	16.5 ± 2.5	24.0 ± 3.5	10.6 ± 1.5
**5**	2.5 ± 0.3	12.0 ± 1.7	30.0 ± 3.5	12.2 ± 1.6

Data are shown as mean values ± SD of three independent determinations performed in triplicate. ND, not determined.
