# Supplementary material for: New Polyether Triterpenoids from Laurencia viridis and Their Biological Evaluation
Source: Mar Drugs. 2011 Nov 7;9(11):2220–35. doi: 10.3390/md9112220 (PMC3229232; doi:10.3390/md9112220)

Supporting Information

New Polyether Triterpenoids from *Laurencia viridis* and their Biological Evaluation

Francisco Cen Pacheco[1], Janny A. Villa-Pulgarin[2,3], Faustino Mollinedo[2], Manuel Norte Martín[1*], José Javier Fernández[1*] and Antonio Hernández Daranas[1*]

1 Instituto Universitario de Bio-Orgánica "Antonio González" (IUBO), Universidad de La Laguna (ULL), Astrofísico Francisco Sánchez 2, 38206 La Laguna, Tenerife, Spain

2 Instituto de Biología Molecular y Celular del Cáncer, Centro de Investigación del Cáncer, CSIC-Universidad de Salamanca, Campus Miguel de Unamuno, E-37007 Salamanca, Spain

3 APOINTECH, Centro Hispano-Luso de Investigaciones Agrarias (CIALE), Parque Científico de la Universidad de Salamanca, C/ Rio Duero 12, E-37185 Villamayor, Salamanca, Spain

INDEX

**Scheme S1**	Isolation scheme for compounds **2**-**5**.	S2
**Figure S1**	^{1}H-NMR spectrum of iubol (**2**).	S3
**Figure S2**	COSY spectrum of iubol (**2**).	S4
**Figure S3**	HSQC spectrum of iubol (**2**).	S5
**Figure S4**	HMBC spectrum of iubol (**2**).	S6
**Figure S5**	NOESY spectrum of iubol (**2**).	S7
**Figure S6**	1D-NOE spectrum of iubol (**2**).	S8
**Figure S7**	^{1}H-NMR spectrum of 22-hydroxy-15(28)-dehydrovenustatriol (**3**).	S9
**Figure S8**	COSY spectrum of 22-hydroxy-15(28)-dehydrovenustatriol (**3**).	S10
**Figure S9**	HSQC spectrum of 22-hydroxy-15(28)-dehydrovenustatriol (**3**).	S11
**Figure S10**	HMBC spectrum of 22-hydroxy-15(28)-dehydrovenustatriol (**3**).	S12
**Figure S11**	NOESY spectrum of 22-hydroxy-15(28)-dehydrovenustatriol (**3**).	S13
**Figure S12**	^{1}H-NMR spectrum of 1,2 dehydropseudodehydrothyrsiferol (**4**).	S14
**Figure S13**	COSY spectrum of 1,2 dehydropseudodehydrothyrsiferol (**4**).	S15
**Figure S14**	Edited HSQC spectrum of **4**.	S16
**Figure S15**	HMBC spectrum of 1,2 dehydropseudodehydrothyrsiferol (**4**).	S17
**Figure S16**	NOESY spectrum of 1,2 dehydropseudodehydrothyrsiferol (**4**).	S18
**Figure S17**	^{1}H-NMR spectrum of secodehydrothyrsiferol (**5**).	S19
**Figure S18**	COSY spectrum of secodehydrothyrsiferol (**5**).	S20
**Figure S19**	Edited HSQC spectrum of secodehydrothyrsiferol (**5**).	S21
**Figure S20**	HMBC spectrum of secodehydrothyrsiferol (**5**).	S22
**Figure S21**	NOESY spectrum of secodehydrothyrsiferol (**5**).	S23

Scheme S1. Isolation scheme for compounds **2**-**5**.

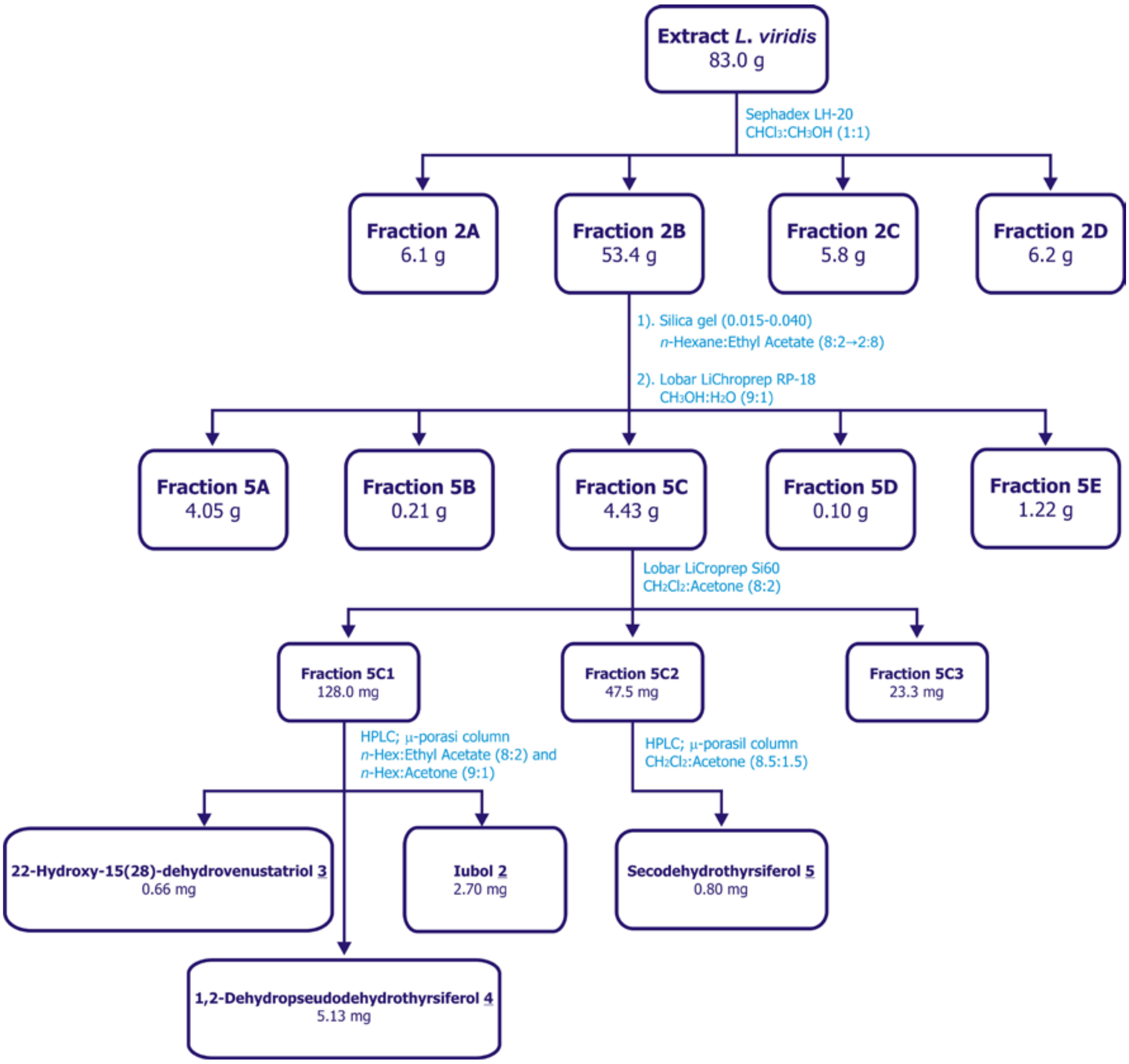

Figure S1. ^{1}H-NMR spectrum of iubol (**2**) (600 MHz; $CDCl_3$; 298 K).

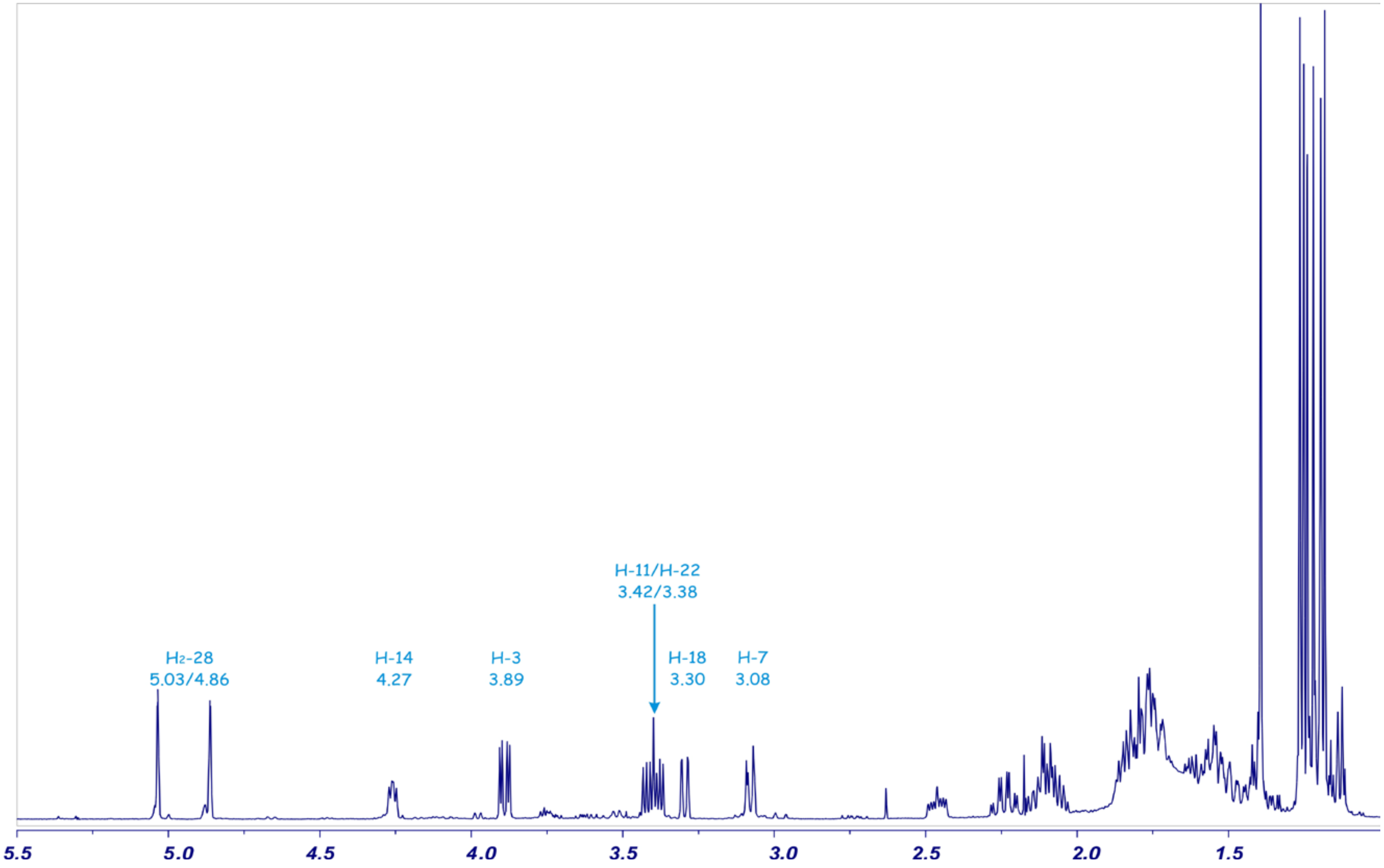

Figure S2. COSY spectrum of iubol (**2**) (600 MHz; $CDCl_3$; 298 K).

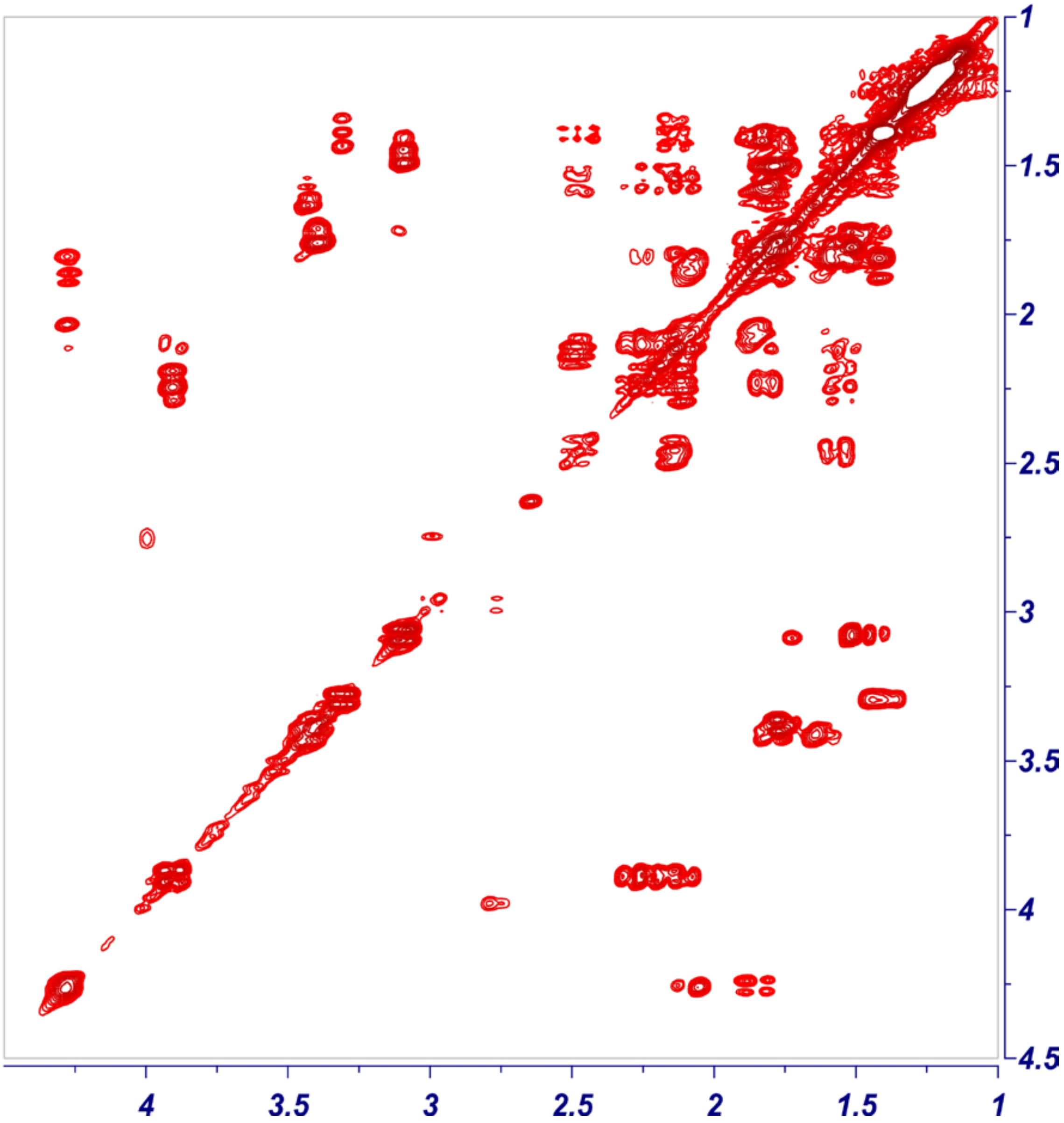

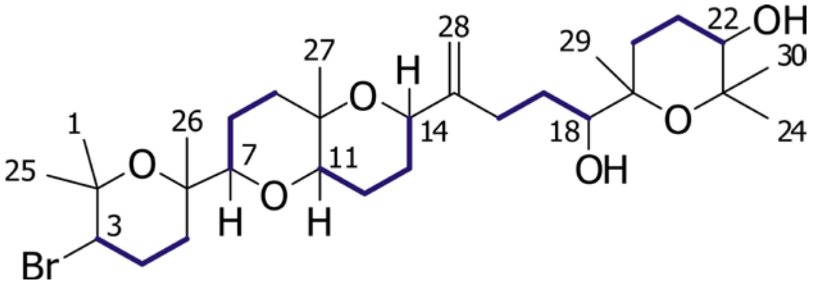

Figure S3. HSQC spectrum of iubol (**2**) (600 MHz; $CDCl_3$; 298 K).

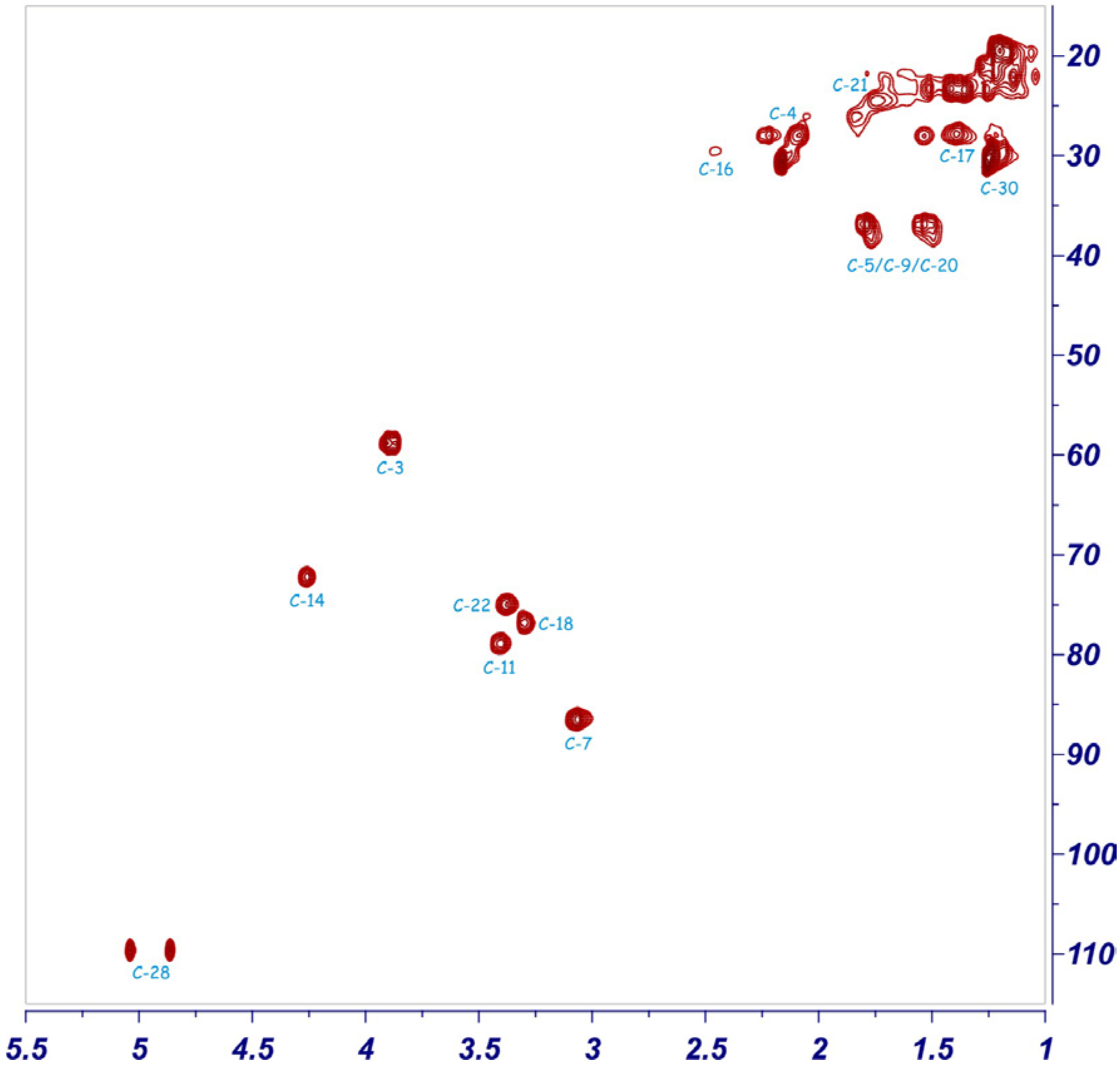

Figure S4. HMBC spectrum of iubol (**2**) (600 MHz; $CDCl_3$; 298 K).

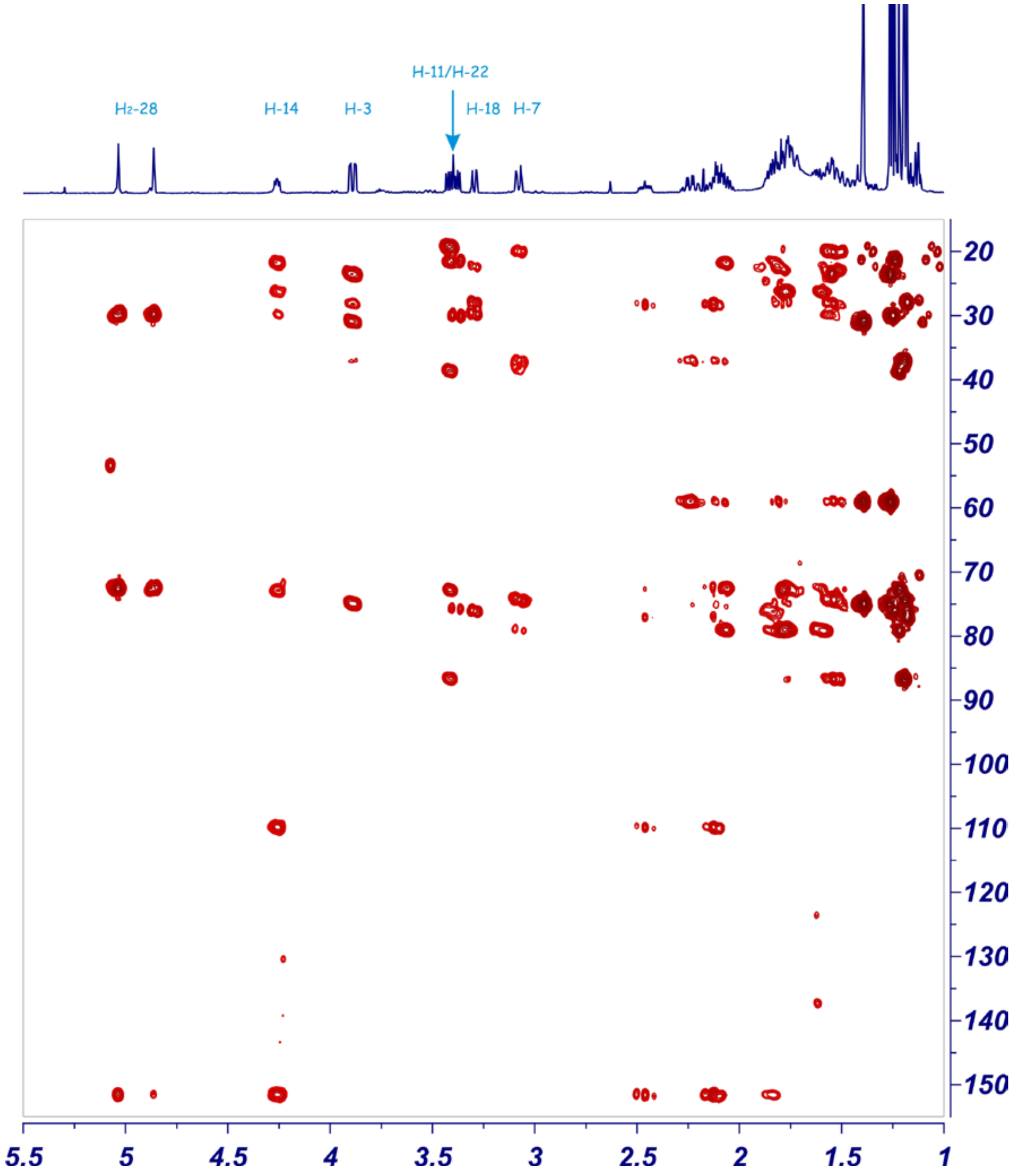

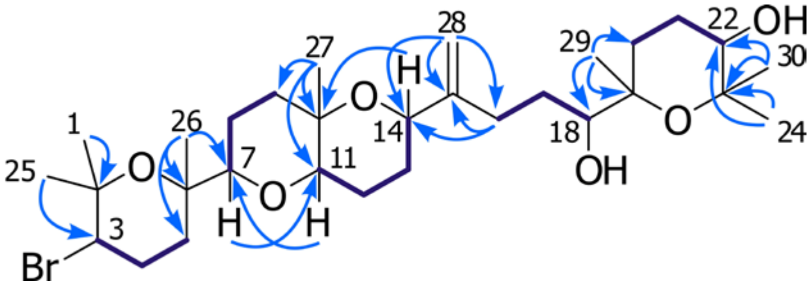

Figure S5. NOESY spectrum of iubol (**2**) (600 MHz; $CDCl_3$; 298 K).

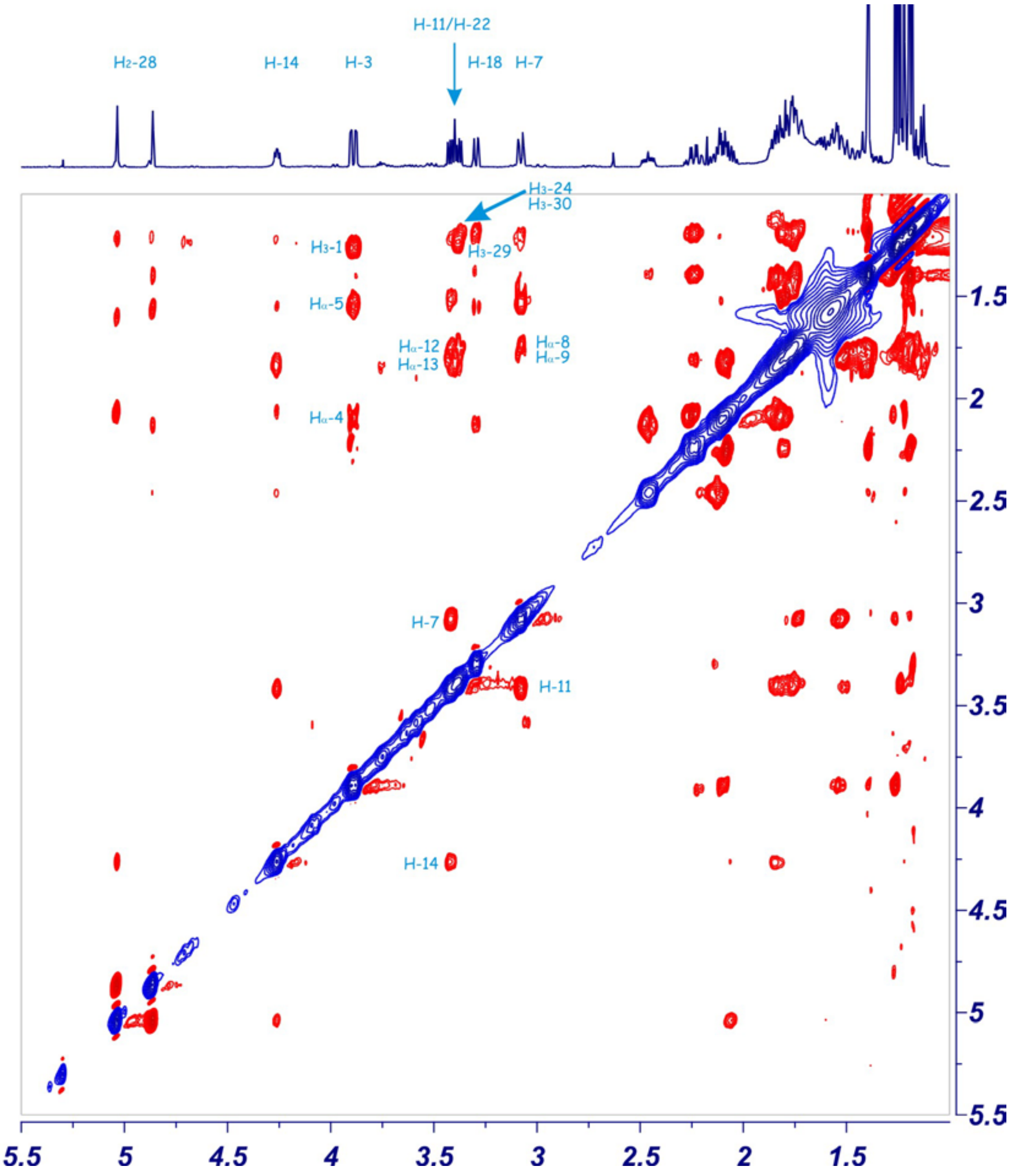

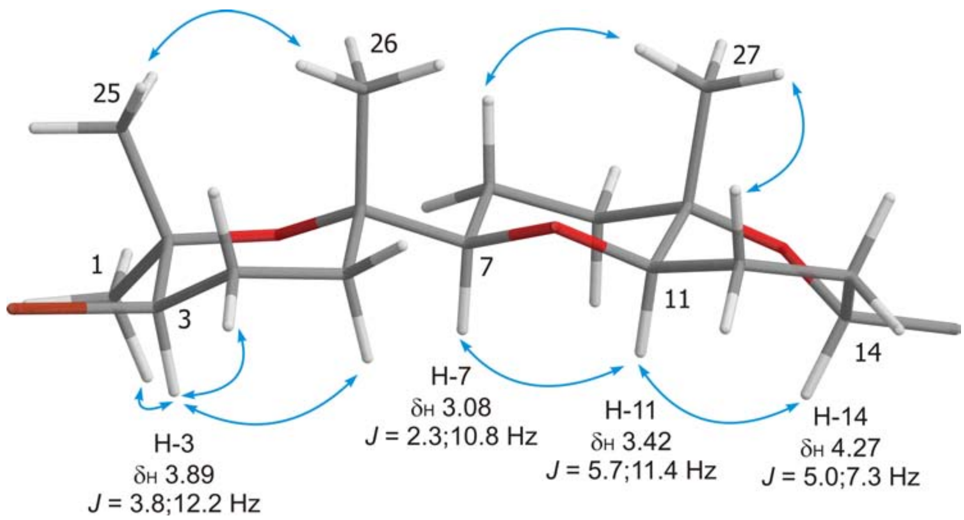

Figure S6. 1D-NOE spectrum of iubol (**2**) (600 MHz; $CDCl_3$; 298 K).

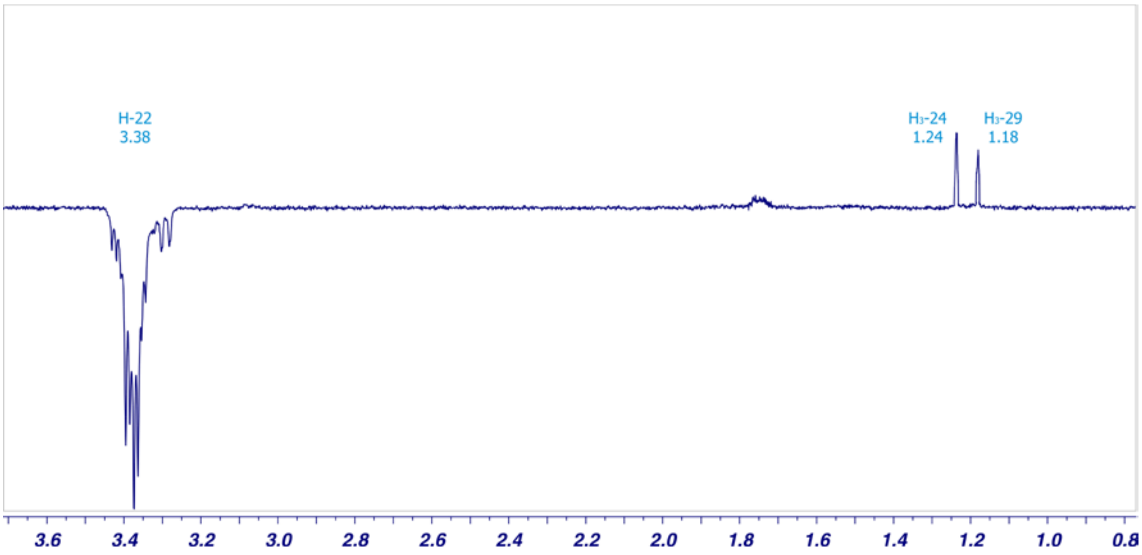

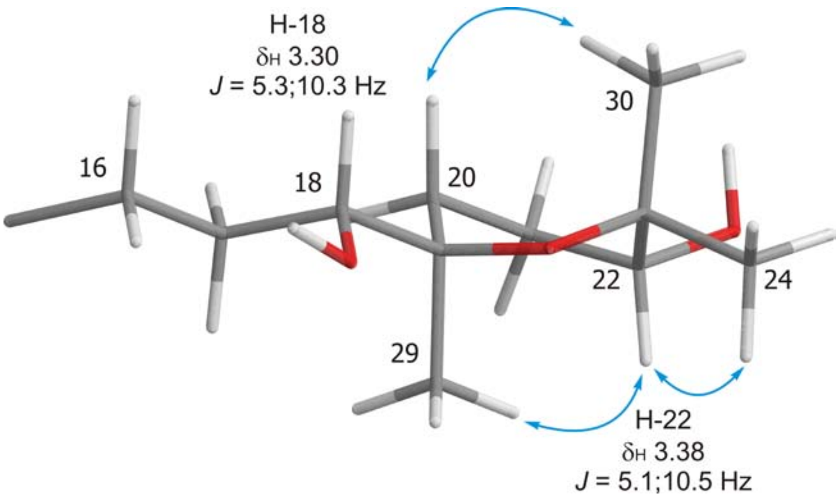

Figure S7. ^{1}H-NMR spectrum of 22-hydroxy-15(28)-dehydrovenustatriol (**3**) (600MHz; $CDCl_3$; 298 K).

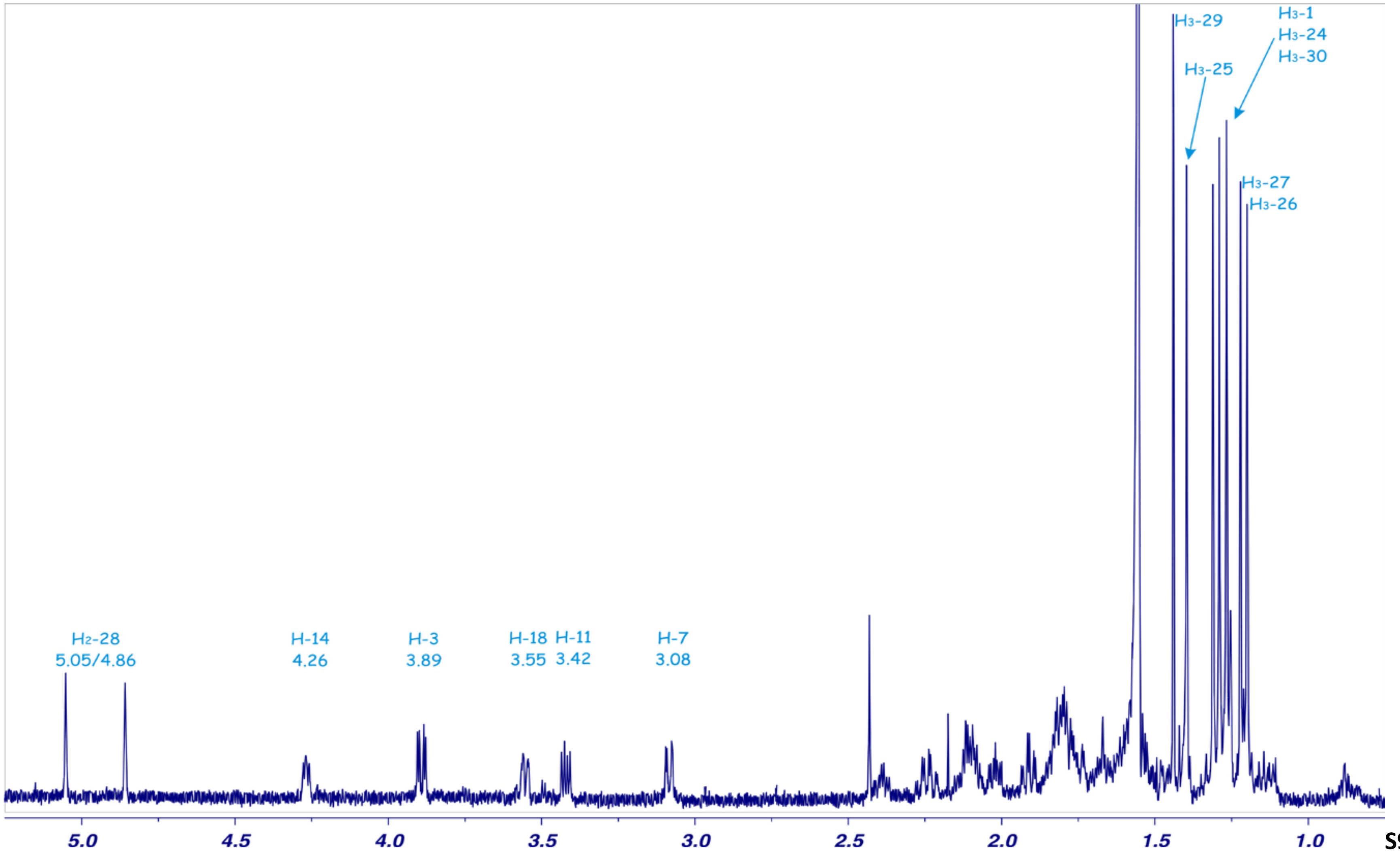

Figure S8. COSY spectrum of 22-hydroxy-15(28)-dehydrovenustatriol (**3**) (600 MHz; $CDCl_3$; 298 K).

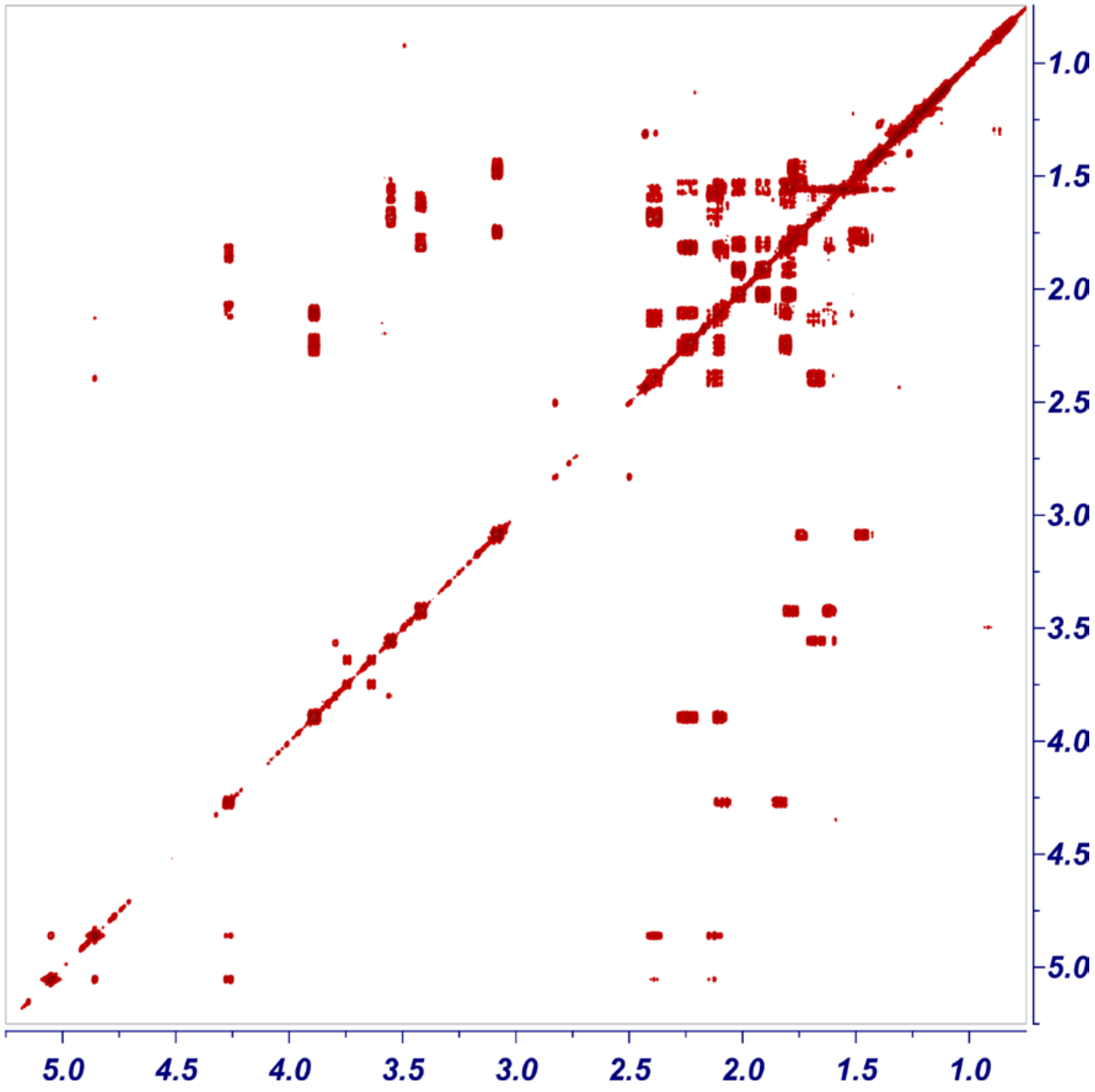

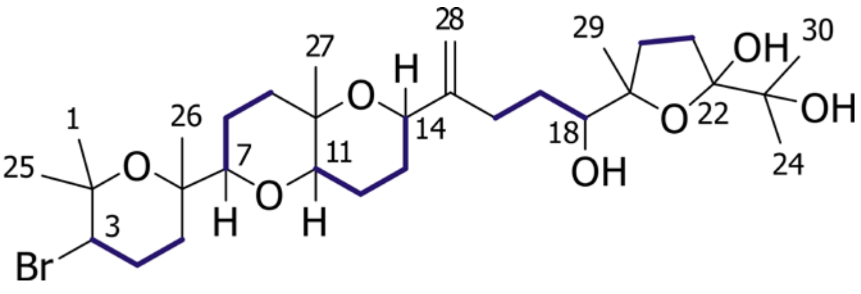

Figure S9. HSQC spectrum of 22-hydroxy-15(28)-dehydrovenustatriol (**3**) (600 MHz; $CDCl_3$; 298 K).

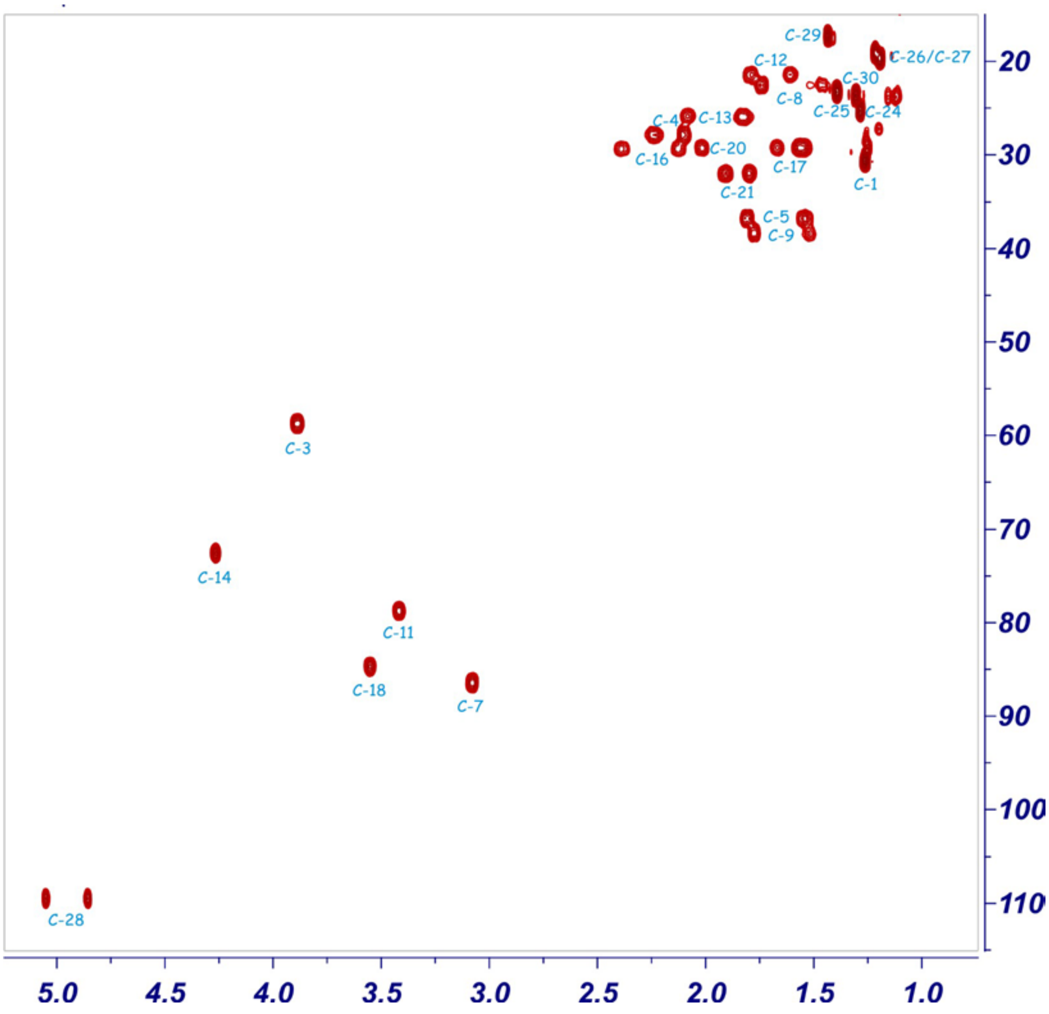

Figure S10. HMBC spectrum of 22-hydroxy-15(28)-dehydrovenustatriol (**3**) (600 MHz; $CDCl_3$; 298 K).

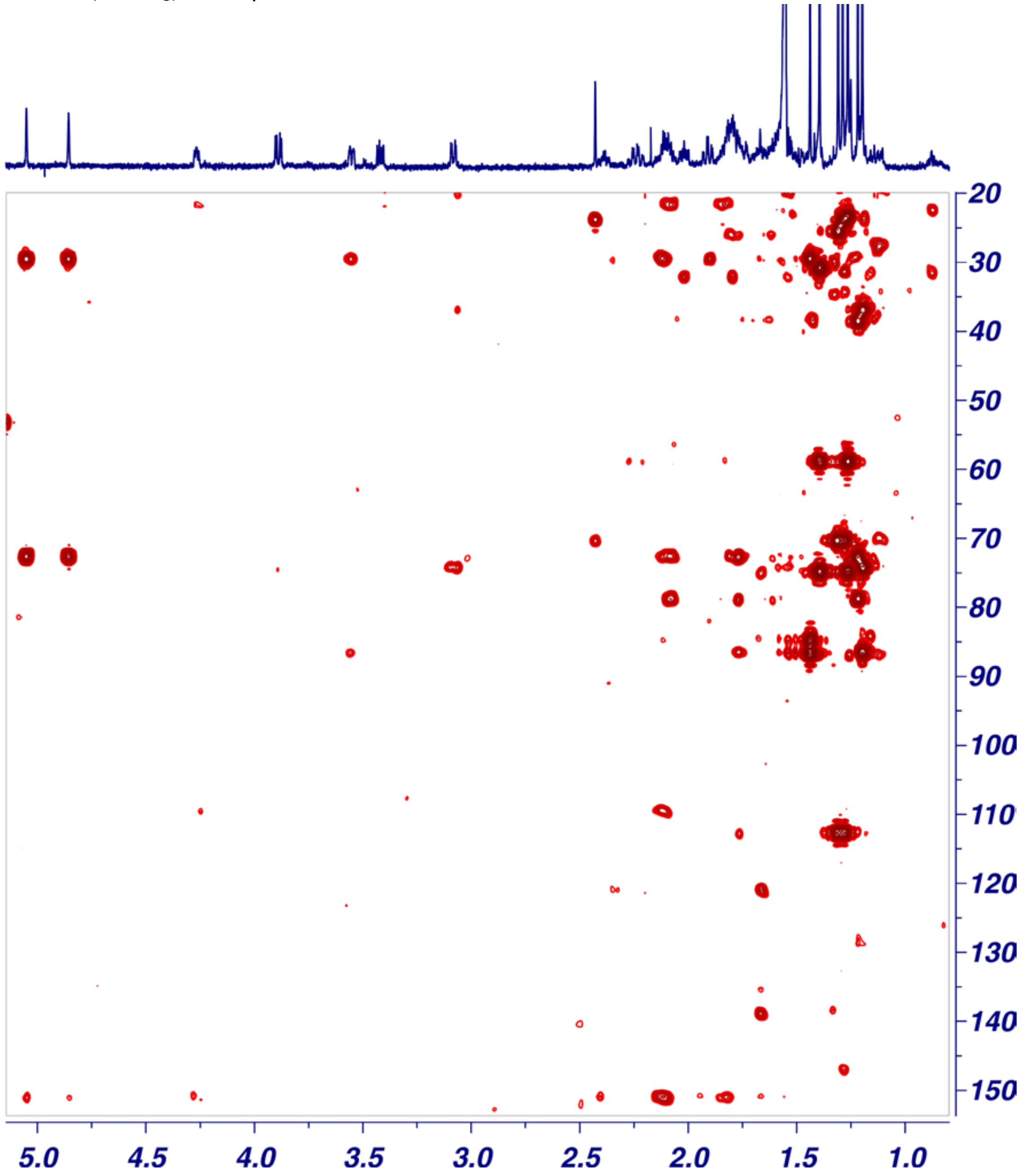

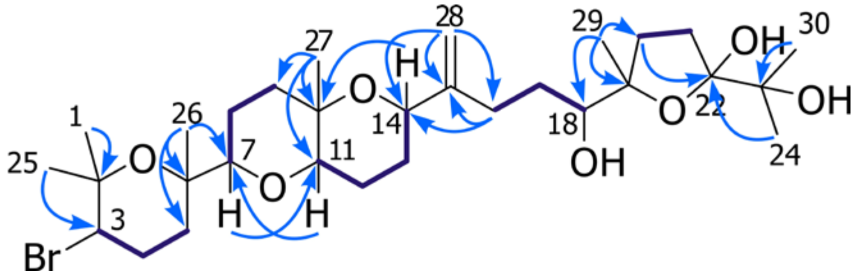

Figure S11. ROESY spectrum of 22-hydroxy-15(28)-dehydrovenustatriol (**3**) (600 MHz; $CDCl_3$; 298 K).

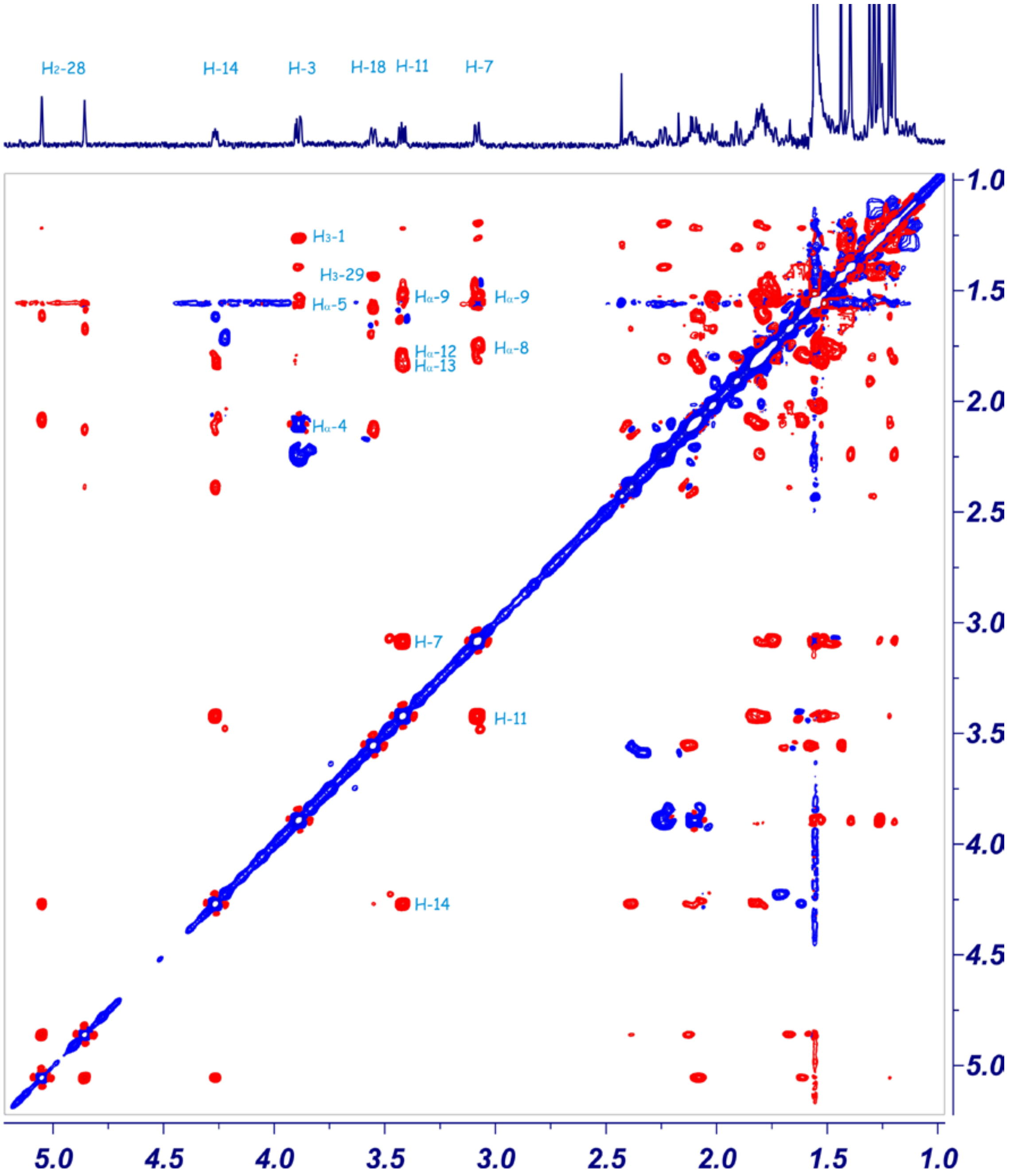

Figure S12. ^{1}H-NMR spectrum of 1,2 dehydropseudodehydrothyrsiferol (**4**) (600 MHz; $CDCl_3$; 298 K).

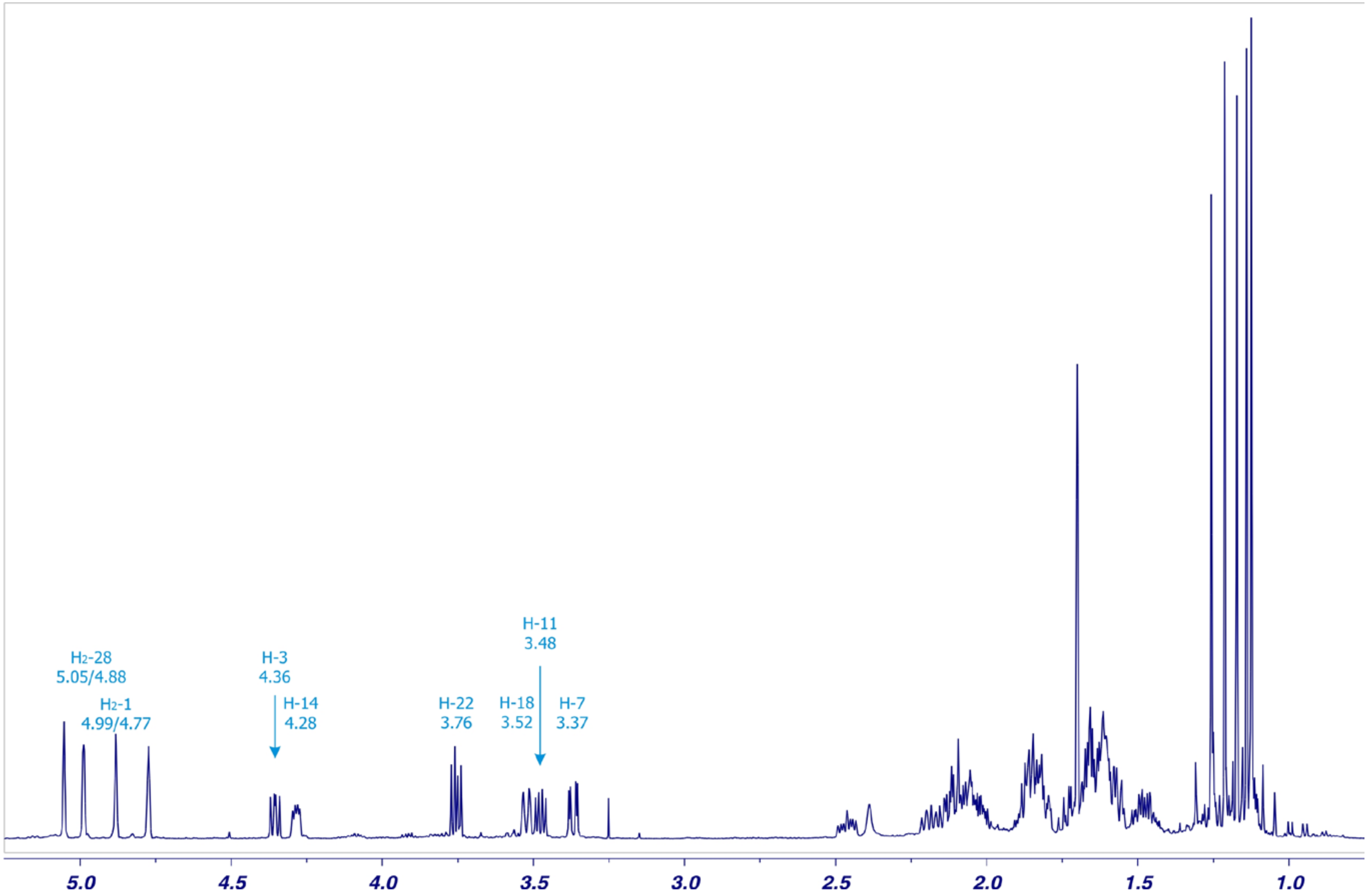

Figure S13. COSY spectrum of 1,2 dehydropseudodehydrothyrsiferol (**4**) (600 MHz; $CDCl_3$; 298 K).

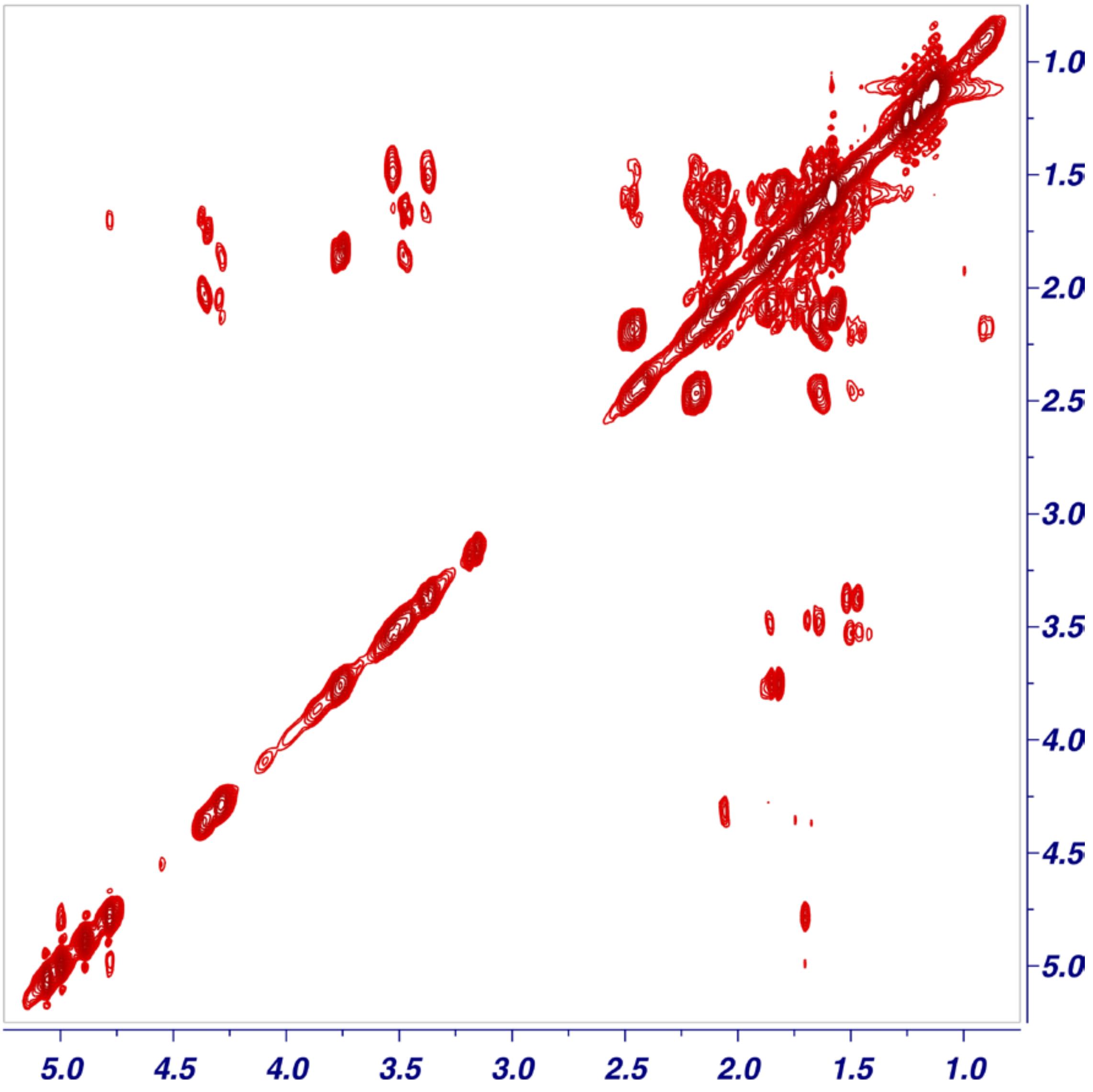

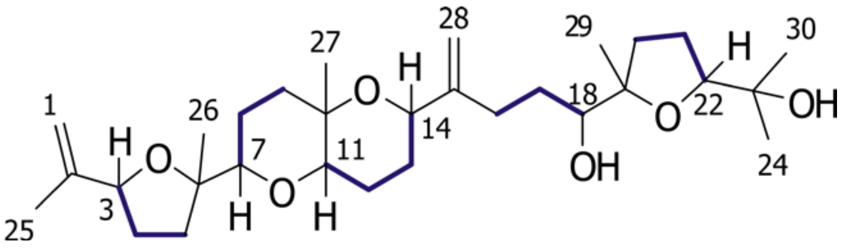

Figure S14. Edited HSQC spectrum of 1,2 dehydropseudodehydrothyrsiferol (**4**) (600 MHz; $CDCl_3$; 298 K).

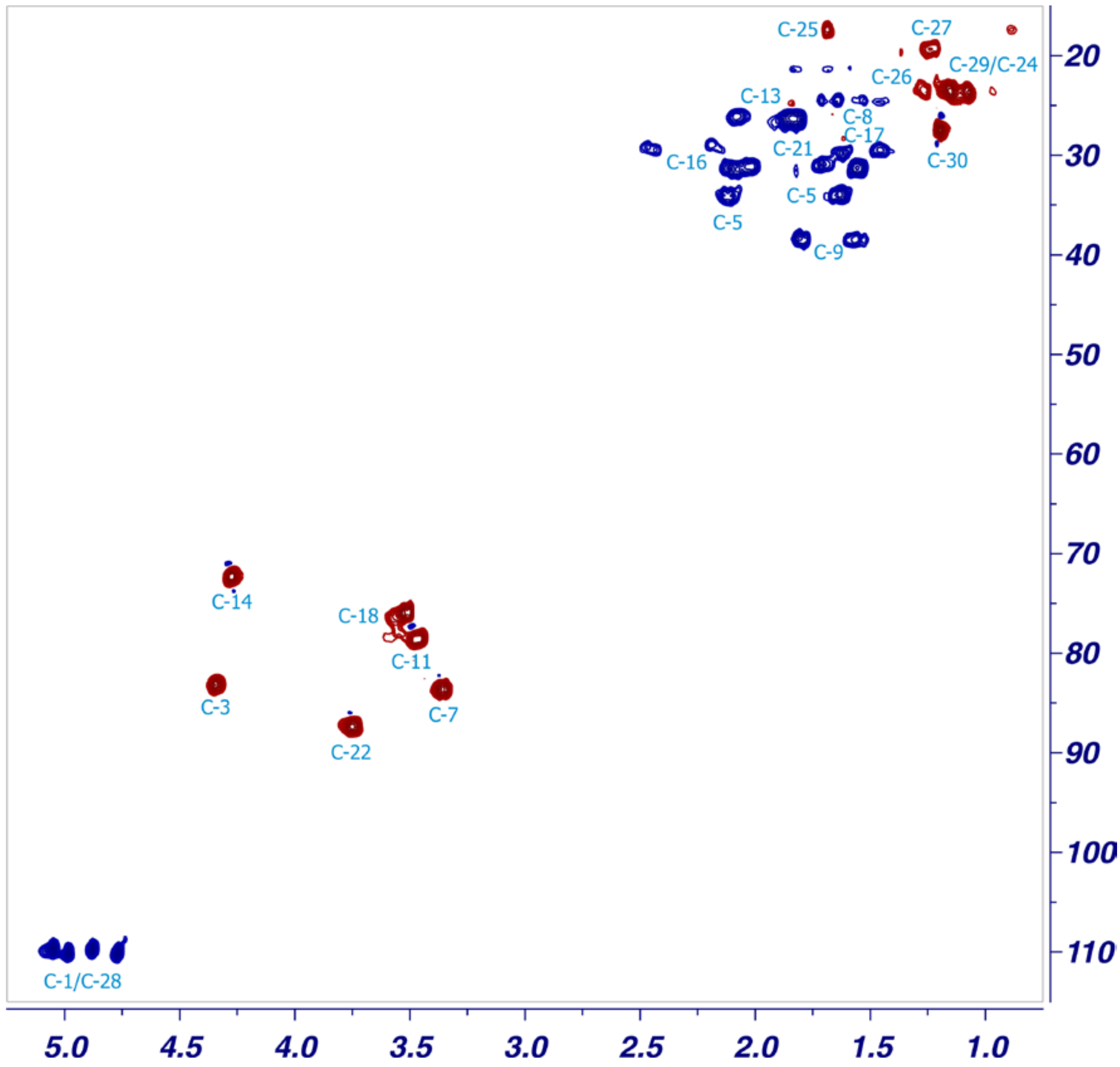

Figure S15. HMBC spectrum of 1,2 dehydropseudodehydrothyrsiferol (**4**) (600 MHz; $CDCl_3$; 298 K).

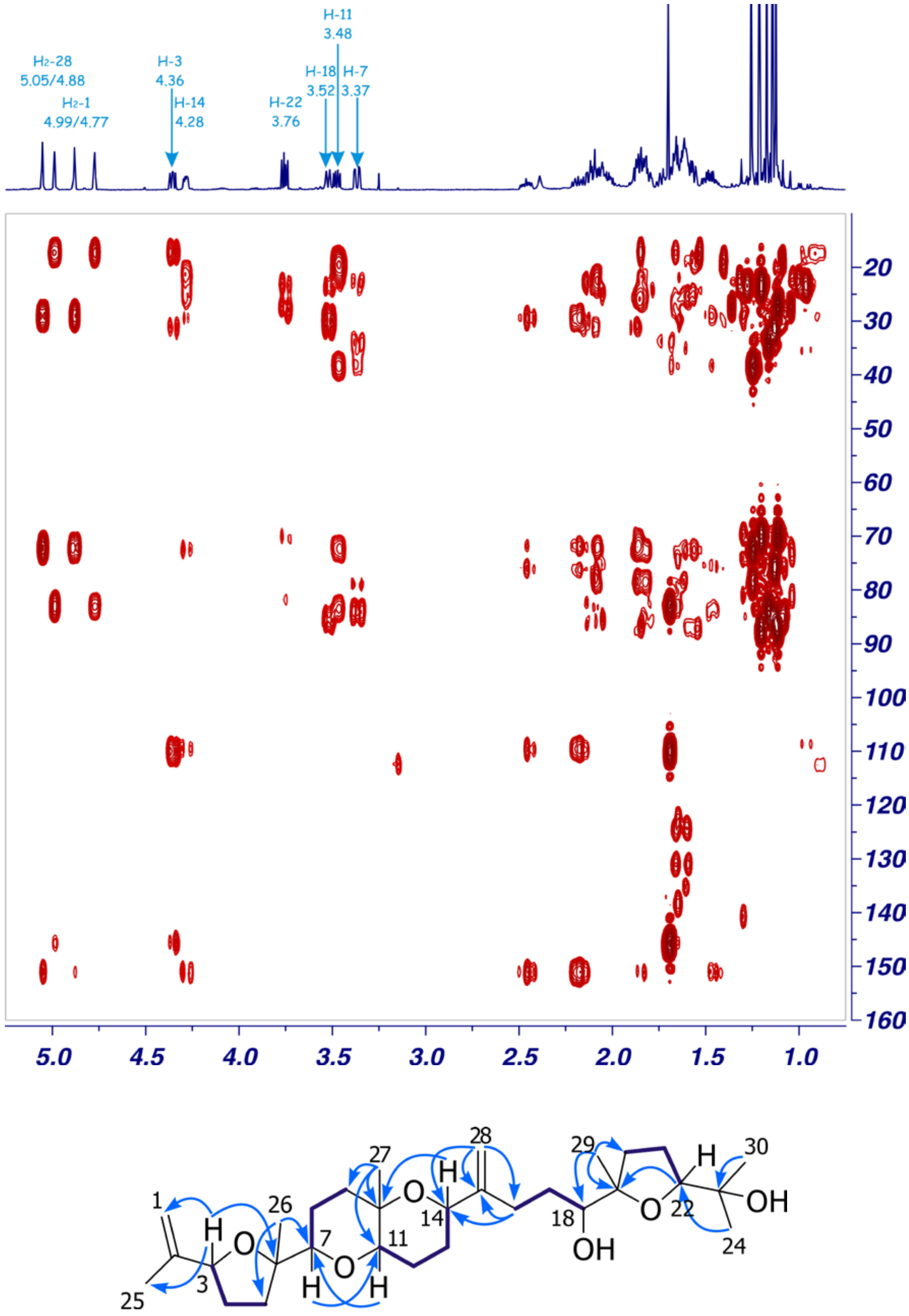

Figure S16. NOESY spectrum of 1,2 dehydropseudodehydrothyrsiferol (**4**) (600 MHz; $CDCl_3$; 298 K).

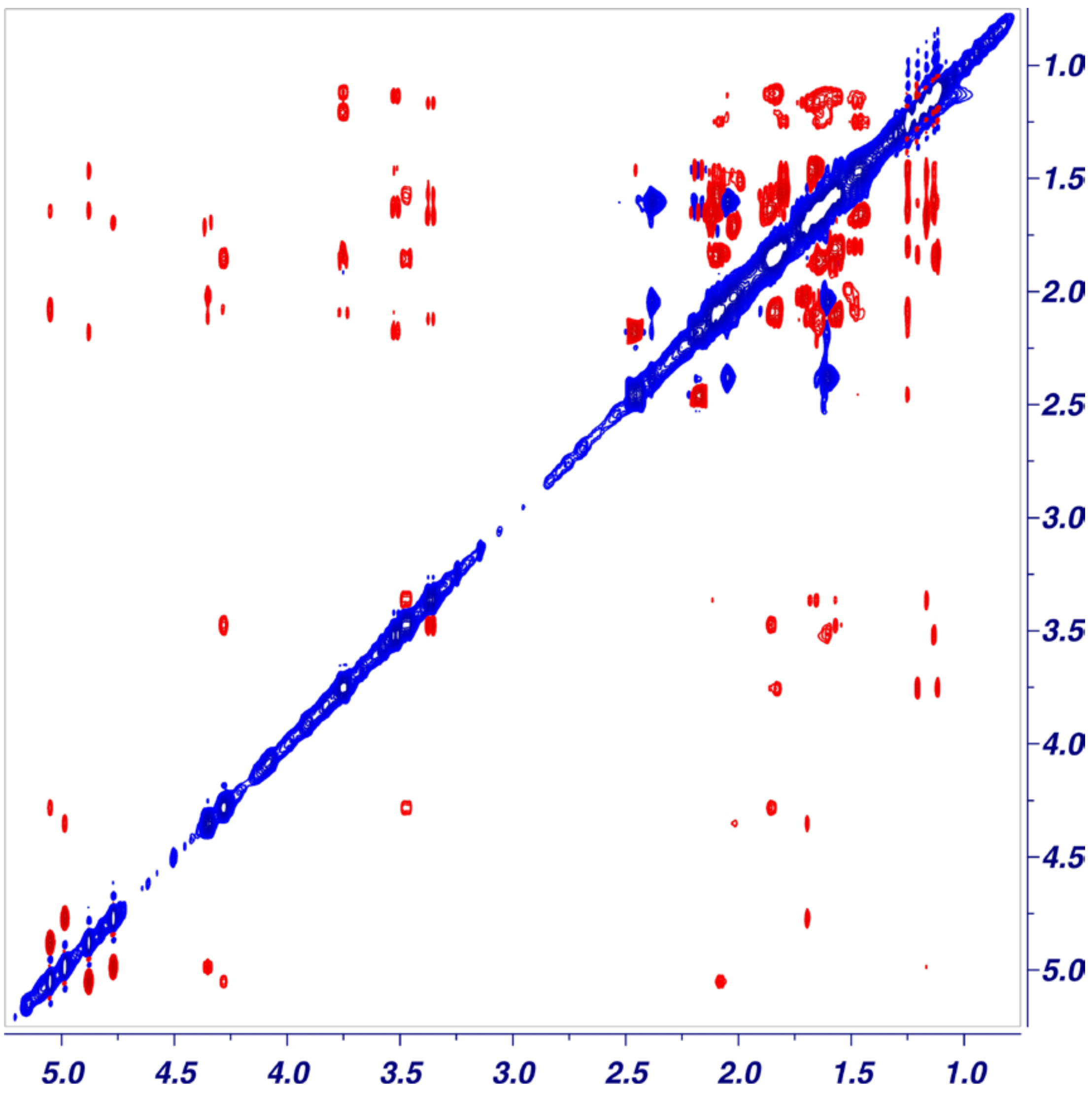

Figure S17. ^{1}H-NMR spectrum of secodehydrothyrsiferol (**5**) (600 MHz; $CDCl_3$; 298 K).

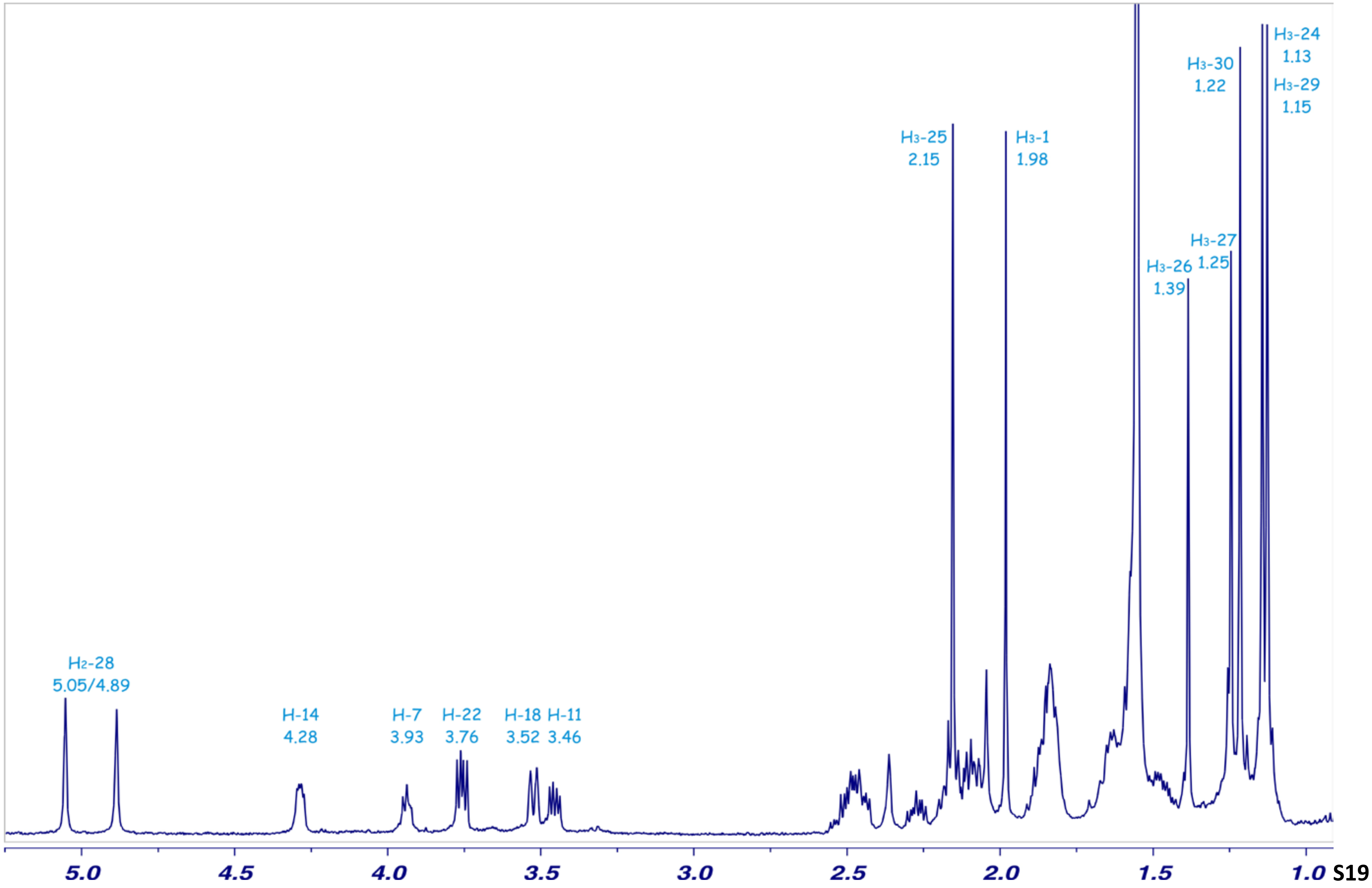

Figure S18. COSY spectrum of secodehydrothyrsiferol (**5**) (600 MHz; $CDCl_3$; 298 K).

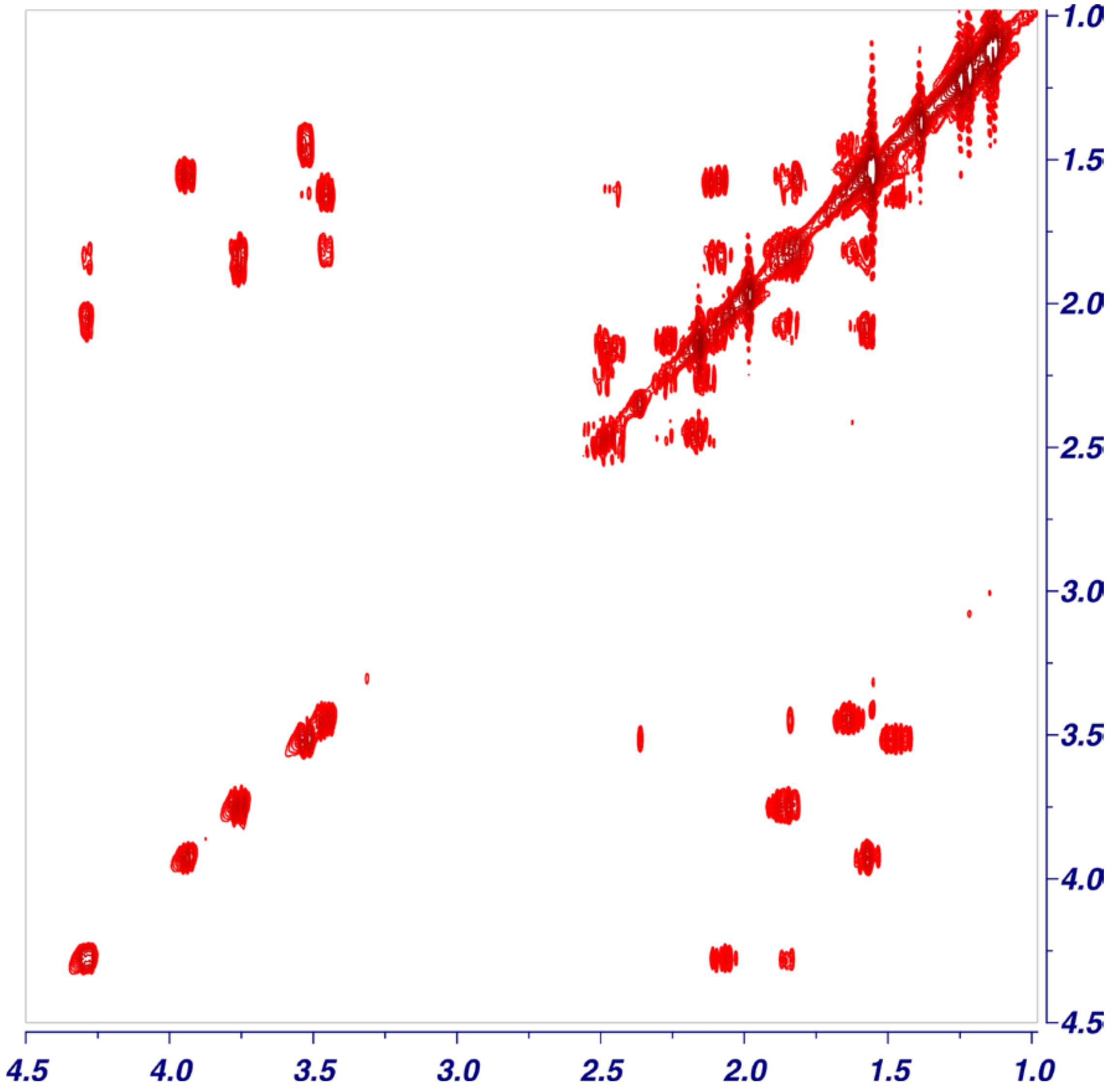

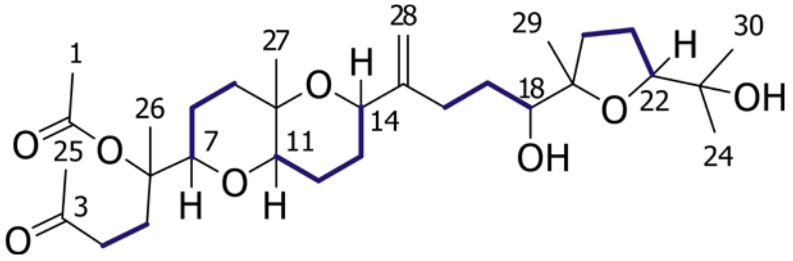

Figure S19. Edited HSQC spectrum of secodehydrothyrsiferol (**5**) (600 MHz; $CDCl_3$; 298 K).

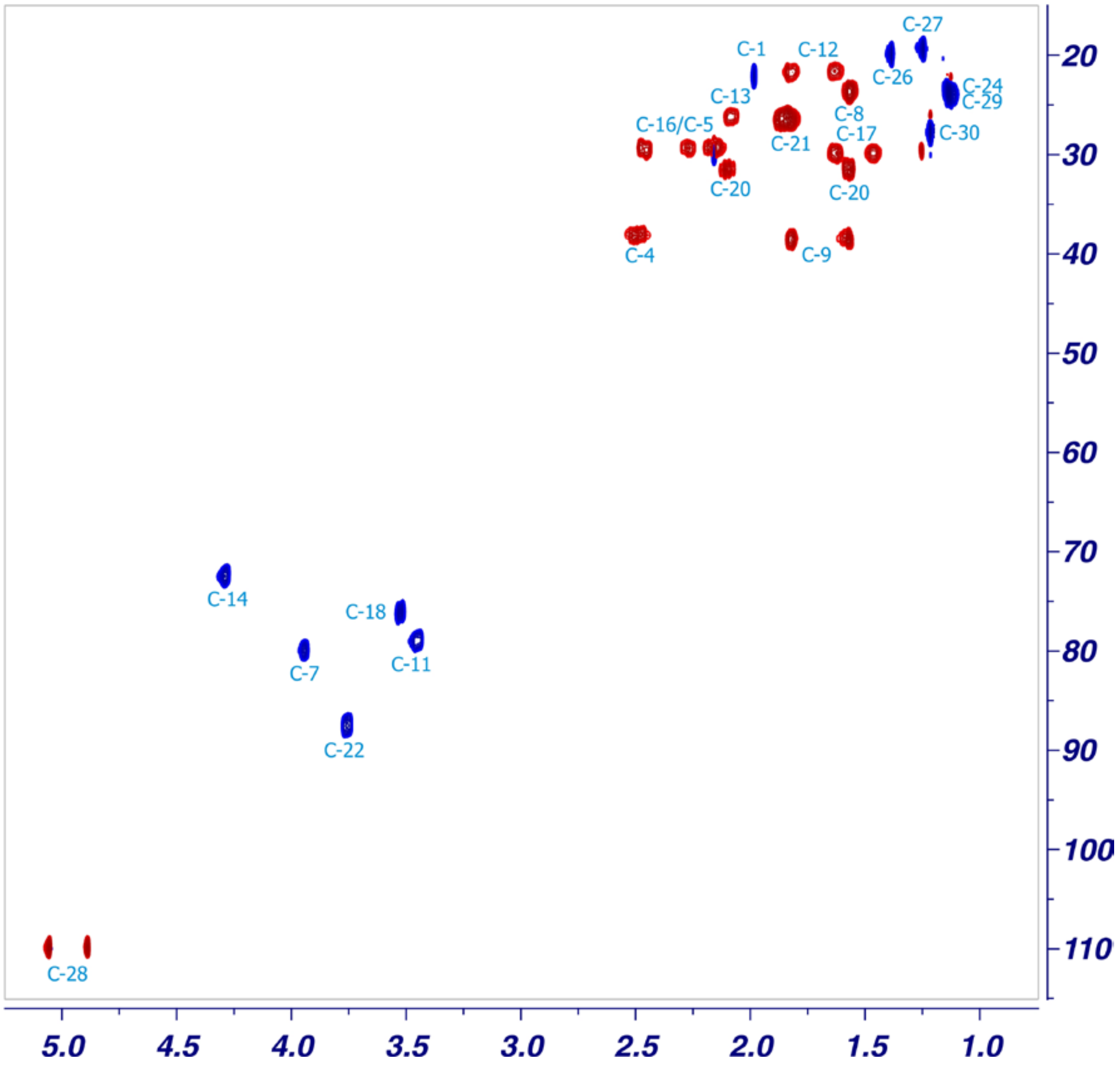

Figure S20. HMBC spectrum of secodehydrothyrsiferol (**5**) (600 MHz; $CDCl_3$; 298 K).

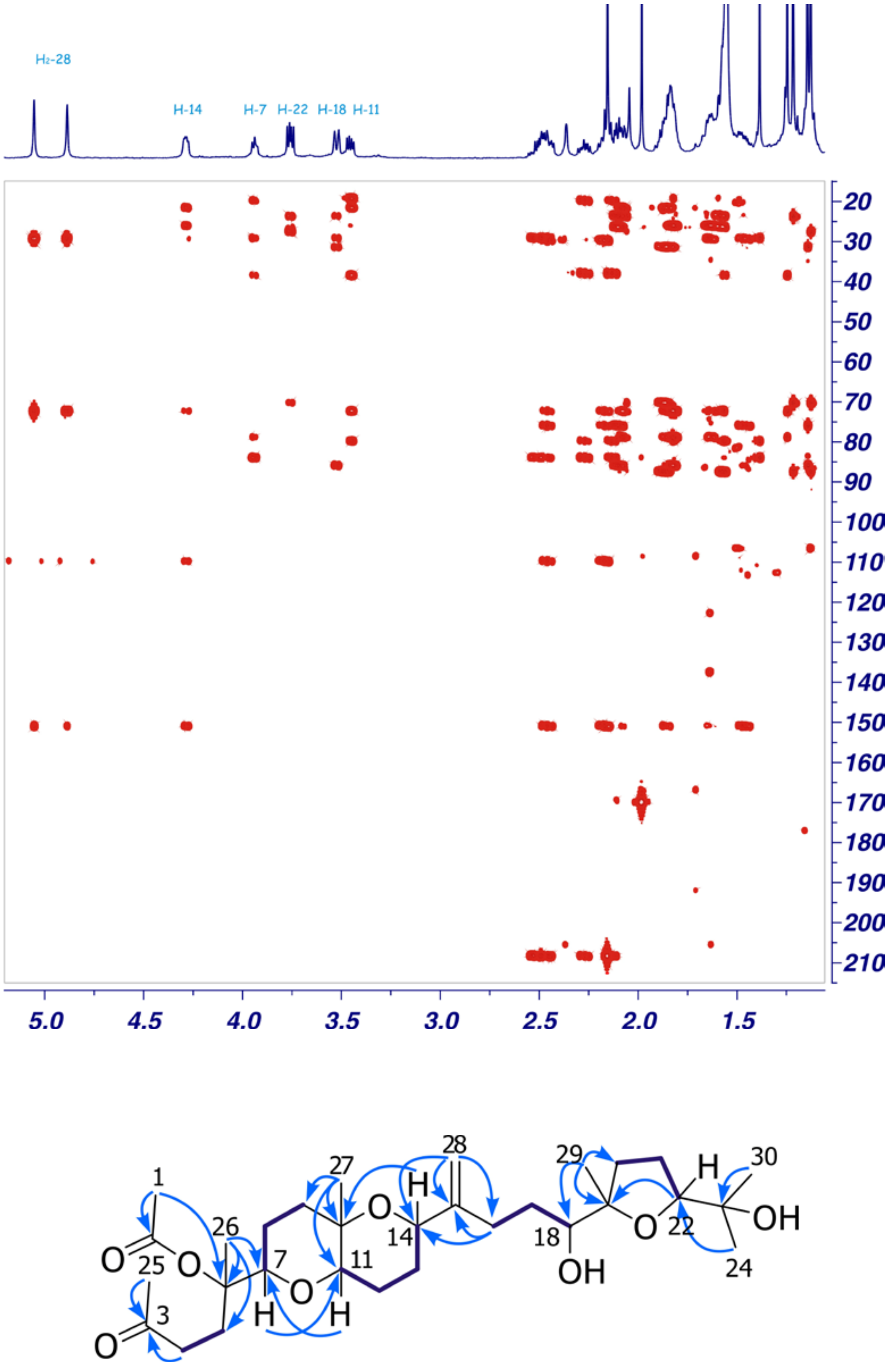

Figure S21. NOESY spectrum of secodehydrothyrsiferol (**5**) (600 MHz; $CDCl_3$; 298 K).

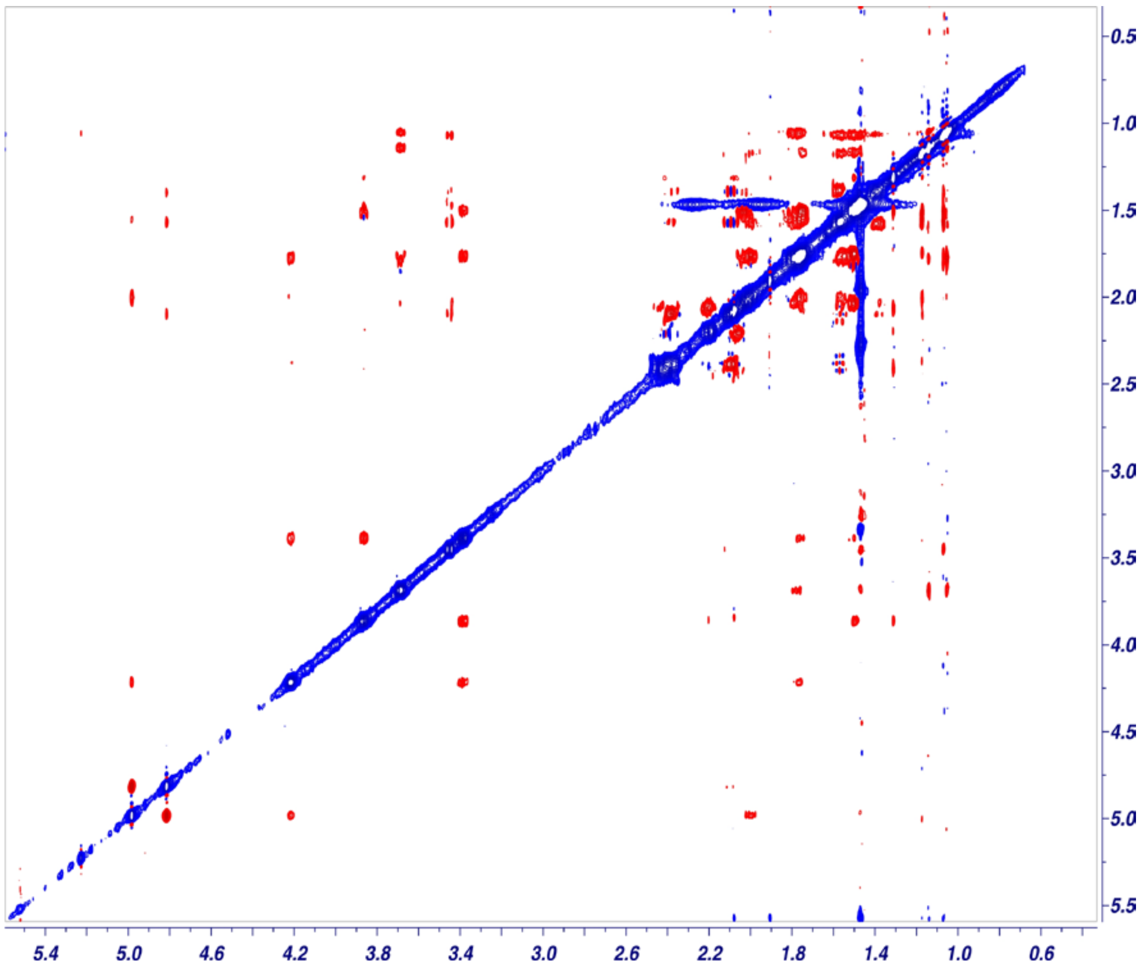

Supplement: Supplementary file 1 [file marinedrugs-09-02220-s001.pdf]
